# Substrate cycles in *Penicillium chrysogenum* quantified by isotopic non-stationary flux analysis

**DOI:** 10.1186/1475-2859-11-140

**Published:** 2012-10-25

**Authors:** Zheng Zhao, Angela ten Pierick, Lodewijk de Jonge, Joseph J Heijnen, S Aljoscha Wahl

**Affiliations:** 1 Department of Biotechnology, Delft University of Technology, Julianalaan 67, Delft, 2628 BC, Netherlands; 2 Current address: DSM Biotechnology Center, Alexander Fleminglaan 1, Delft, 2613 AX, Netherlands; 3 Department of Biotechnology, Kluyver Centre for Genomics of Industrial Fermentation, Delft University of Technology, Julianalaan 67, Delft, 2628 BC, Netherlands

## Abstract

**Background:**

*Penicillium chrysogenum*, the main production strain for penicillin-G, has a high content of intracellular carbohydrates, especially reduced sugars such as mannitol, arabitol, erythritol, as well as trehalose and glycogen. In previous steady state ^13^C wash-in experiments a delay of labeling enrichments in glycolytic intermediates was observed, which suggests turnover of storage carbohydrates. The turnover of storage pools consumes ATP which is expected to reduce the product yield for energy demanding production pathways like penicillin-G.

**Results:**

In this study, a ^13^C labeling wash-in experiment of 1 hour was performed to systematically quantify the intracellular flux distribution including eight substrate cycles. The experiments were performed using a mixed carbon source of 85% Cmol_Glc_/Cmol_Glc+EtOH_ labeled glucose (mixture of 90% [1-^13^C_1_] and 10% [U-^13^C_6_]) and 15% ethanol [U-^13^C_2_]. It was found, that (1) also several extracellular pools are enriched with ^13^C labeling rapidly (trehalose, mannitol, and others), (2) the intra- to extracellular metabolite concentration ratios were comparable for a large set of metabolites while for some carbohydrates (mannitol, trehalose, and glucose) the measured ratios were much higher.

**Conclusions:**

The fast enrichment of several extracellular carbohydrates and a concentration ratio higher than the ratio expected from cell lysis (2%) indicate active (e.g. ATP consuming) transport cycles over the cellular membrane. The flux estimation indicates, that substrate cycles account for about 52% of the gap in the ATP balance based on metabolic flux analysis.

## Background

Substrate cycles are metabolic cycles that result in ATP-consumption without net substrate-to-product conversion. These cycles are found in a wide range of organisms, including prokaryotes [1], plants [2], insects [3], and mammalian cells [4].

Some of these cycles are considered to play important physiological roles, such as heat generation [5], metabolic control, and increased metabolic flexibility [6]. These early studies mainly led to qualitative or assumption-based quantitative information on the observed substrate cycle rates. For example, Clark *et al.*[4] indicated activities of glucose-G6P cycle and F6P-FBP cycle in liver cells with the help of [2,5-^3^H _2_, U-^14^C _6_ glucose. However, the validity of their assumptions was seriously questioned by Hue and Hers [7] and Rognstad and Katz [8]. Although Rognstad and Katz [8] attempted to introduce a mathematical model to quantify the F6P-FBP cycle, several additional assumptions were required due to the lack of quantitative knowledge on the exchange fluxes.

Rigorous mathematical modeling of the isotopic distributions has substantially matured in the last two decades [9-11]. These developments enabled more accurate quantification of fluxes, including substrate cycles. For example, Dauner *et al.*[1] estimated the anaplerotic reactions between PEP-PYR-OAA in *Bacillus subtilis* using a steady-state ^13^C modeling approach proposed by Schmidt *et al.*[12]. Recently Alonso *et al.*[13] also demonstrated the significance of the exchange between glucose-G6P as well as F6P-FBP for the ATP metabolism in maize seeds.

Although the approach of steady-state ^13^C flux analysis used in these works require less assumptions compared to stoichiometric analysis, a few drawbacks still remain. One of them is the requirement of an isotopic steady state for metabolites resp. proteinogenic amino acids. This not only results in a long labeling time, but also valuable information of the labeling transients are not used [14-16]. Especially Nöh *et al.*[16] have shown theoretically, that istotopic non-stationary state flux analysis increases the accuracy of the flux estimation. Noack *et al.*[17] compared the results from non-stationary and stationary state ^13^C flux analysis based on a comparable data-set for *C. glutamicum* and showed that the INST approach delivers more accurate and reasonable data. Flux analysis based on the isotopic non-stationary data (INST) emerged as new tool for systems biology studies in recent years [18,19]. The INST approach requires high computational efforts [16] and especially for a large model, large amounts of differential equations have to be solved. To our knowledge, the largest INST metabolic network published so far contains 86 reactions (for *E. coli*, ref. [19]). The computational demands further increase when cellular compartmentation is considered (metabolites present in different compartments have separate balances).

In this study the flux distribution of *P. chrysogenum* was quantified using a large scale INST ^13^C metabolic model. This detailed model considers three different cellular compartments and 20 intracellular transport reactions. In total, 177 metabolic reactions and 94 pools were included.

In the experiment, a substrate mixture of glucose (85% Cmol_Glc_/Cmol_Glc+EtOH_) and ethanol (15% Cmol_EtOH_/Cmol_Glc+EtOH_) was used as limiting carbon-source. This substrate mixture facilitates the quantification of substrate cycles in lower glycolysis and TCA. The quantified substrate cycle fluxes were further supported by the results from enzyme activity assays.

## Results

### Metabolic flux analysis

The measured biomass dry weight was 6.17 g/L, which is comparable with previous experiments [20,21] under similar conditions. Based on the measured uptake and secretion rates and the stoichiometric metabolic model (Additional file 1: Table S1), the intracellular rates were calculated (Additional file 1: Table S3). The stoichiometric model was also used to calculate the ATP dissimilation by yet unknown processes, which is summarized as maintenance requirements (non-growth-associated, growth-associated, and product-associated). To estimate the value, assumptions on the P/O ratio and ATP demands for biomass synthesis are required. We chose to use the P/O ratio reported in van Gulik *et al.*[22] as the strain used is the same (only the name changed) and the cultivation conditions are comparable (carbon limited chemostat). The requirements for biomass synthesis, e.g. polymerization are the same as described by van Gulik et al. [23]. ATP that is not consumed for the biomass reaction and other balanced processes is ‘sinked’ in reaction r15.1 which reflects the ATP dissimilation in yet unknown processes. Based on the metabolic flux analysis, 82.1 mmol/Cmol_BM_/h ATP were consumed (r15.1 in Additional file 1: Table S3).

### Measured metabolite concentrations

Most central carbon metabolites could be measured in the intracellular space but were also detected at very low quantities in the extracellular filtrate. It can be seen that the ratios of the intracellular to extracellular concentration of all phosphorylated sugars are constant at an average value of 51±5 (IC/EC, Table 1). This consistency is a strong indication of limited cell lysis. Assuming this ratio only results from lysis and all cells contain comparable metabolite levels, approximately 2% of the total cell population lost their membrane integrity and released metabolites. Pyruvate and most carboxylic acids of the TCA cycle have lower IC/EC concentration ratios (except citrate and malate), which indicates either export or leakage during quenching.

**Table 1:** Measured metabolite concentrations and the ratio of intra/extracellular concentrations (assuming a cellular volume of 2.5 mL/gDW)

	**IC**	**IC**	**EC**	**ratio**
	**[μmol/g]**	**[mM]**	**[mM]**	**[−]**
TRE	20.4	8.158	0.0204	401
GLC	0.643	0.257	0.0017	151
ICITR	0.041	0.016	0.0001	123
MANOL	175	69.9	0.580	121
CITR	1.69	0.676	0.0061	110
PG2	0.076	0.030	0.0004	82
PG3	0.870	0.348	0.0049	71
MAL	2.50	1.00	0.0179	56
RIB5P	0.739	0.296	0.0054	55
TRE6P	0.201	0.080	0.0015	55
FBP	0.358	0.143	0.0026	54
RBTOL	4.01	1.60	0.0305	53
M6P	1.01	0.404	0.0077	52
SED7P	2.20	0.881	0.0182	48
F6P	0.675	0.270	0.0062	44
G6P	3.05	1.22	0.0288	42
ERYTOL	9.18	3.67	0.115	32
SUCC	0.631	0.252	0.0151	17
DHAP	0.126	0.051	0.0032	16
FUM	0.773	0.309	0.0457	7
AKG	0.576	0.230	0.0403	6
PYR	0.513	0.205	0.0402	5
E4P	0.021	0.008	0.0000	-
PEP	0.155	0.062	ND	ND
PG6	0.055	0.022	ND	ND
G3P	0.357	0.143	ND	ND

*ND: Not detected.

The intracellular concentration of carbohydrates (Table 1) are about 100–400 times higher than the extracellular concentration. To maintain this large concentration gradient, active uptake of these compounds or extracellular conversion is required. Active uptake, independent of its mechanism, is associated with ATP consumption. E.g. for glucose, this finding is consistent with the described uptake proton symport mechanism [24].

### *A priori* analysis of the labeling dynamics

**Figure 1:**
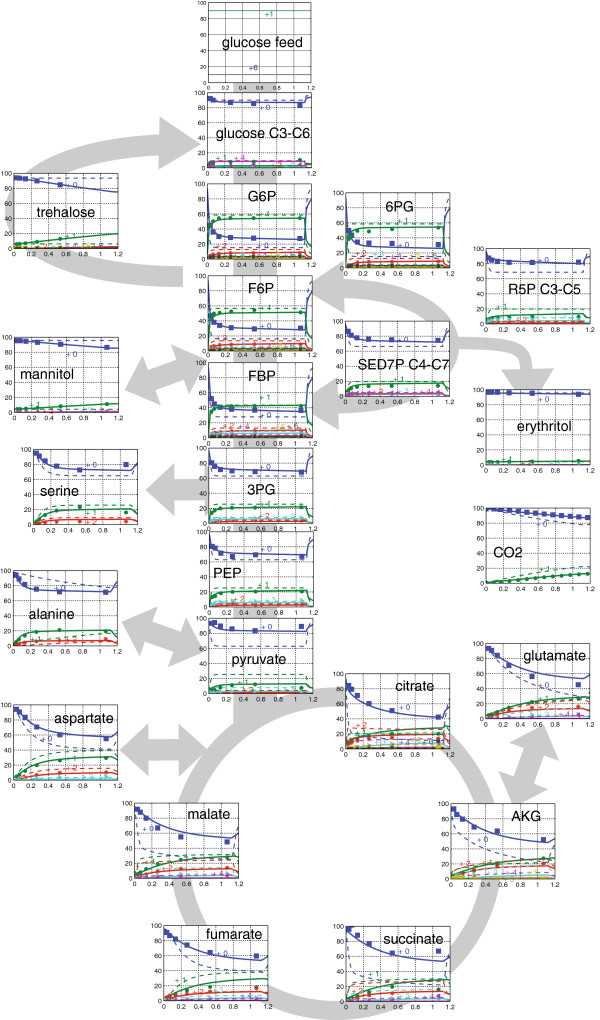
**Mass isotopomer distribution of metabolites after switching to labeled substrate.** Markers are the measured data. The solid line plot is based on the extended metabolic model (after parameter estimation). The dashed lines represent the best fit with the original metabolic model (without substrate cycling).

The dynamics of the measured and corrected for natural mass isotopes mass isotopomer distributions are shown in Figure 1 (and Additional file 2: Table S6). As expected, the enrichments of the intermediates of tricarboxylic acid cycle (TCA) cycle, amino acids, and storage carbohydrates are slower compared to metabolites of glycolysis and pentose phosphate pathway (PPP). However, several unexpected patterns are found, which will be discussed in detail in the following sections.

### The isotopic dynamics of glycolytic intermediates

The glycolytic intermediates (except pyruvate) reached a quasi isotopic steady state after about 10–15 min. This time span is about 60 times longer than expected, considering the typical time constants calculated by the pool sizes (see Table 1) and fluxes (Additional file 1: Table S3) of glycolytic intermediates such as G6P (19 seconds).

Furthermore, the m+1 fraction of the C1-C6 containing glucose-6-phosphate (G6P) measurement only reached about 60% after 1 hour of labeling. This is below the labeling fraction of the labeled substrate, glucose (90% 1-^13^C _1_). Additionally, a m+2 fraction (8.3%) and m+0 fraction (27.5%) which are not present in the labeled glucose feed were observed. While the m+0 fraction indicates an influx of unlabeled carbon, the m+2 fraction indicates carbon rearrangements.

The m+0 can originate from the degradation products of trehalose and glycogen returning to glycolysis via glucose to G6P. Mannitol reenters at F6P. Due to the fast bidirectional reaction of phosphoglucoisomerase (pgi, as evidenced from the nearly identical labeling of F6P and G6P), mannitol and trehalose that were unlabeled at the beginning can contribute to both unlabeled F6P and G6P respectively.

In addition, erythritol and arabitol could slow down the labeling dynamics of the upper glycolysis via their respective precursors in the non-oxidative branch of the pentose phosphate pathway.

### The C3-C6 fragment of G6P

Besides the C1-C6 measurements, the labeling of a C3-C6 fragment of G6P was measured by GC/MS. Deconvolution of the labeling of G6P with the C3-C6 fragment gives an estimation of the C1-C2 fragment (see Supplement). For the sample taken at 64 min, the estimated C1-C2 fragment has an enrichment of 35.3% m+0, 64.7% m+1, and 0.0% m+2. The measured m+2 fraction of the C3-C6 fragment is much lower (3.6%) than the m+1 fraction (12.6%). This indicates that the m+2 fraction in the C1-C6 fragment has two labeled carbons distributed over the carbon atoms in the C1-C2 and the C3-C6 fragment. Since the m+1 fraction on the C3-C6 fragment cannot originate directly from the 1-^13^C labeled glucose feed, it must be a result of metabolic activity. Three alternative routes can explain the m+1 labeling enrichment of the C3-C6 fragment, resp. m+2 in C1-C6:

1. Non-oxidative PPP route: The aldolase reaction converts a C1 labeled fructose-1,6-bisphosphate into C3-labeled dihydroxyacetone (DHAP) (and unlabeled GAP). Via triose-isomerase (TPI) C3-labeled DHAP reacts to C3-labeled glyceraldehydes-phosphate (GAP). In the transaldolase reaction, sedoheptulose-7-phosphate (S7P) and GAP can produce a C6 labeled F6P. This eventually results in a C6 labeled G6P due to the high reversibility of phosphoglucoisomerase.

2. FBPase route: C3-labeled GAP (and DHAP via TPI) can produce C1 and/or C6-labeled FBP via fructose-bisphosphatealdolase (assuming fast exchange in TPI, which can result in 20% FBP labeled on C1, 20% FBP labeled on C6, and 8% FBP labeled on both C1 and C6). The labeled carbon can thus be delivered to C6 (and C1+C6) in F6P by FBPase activity.

3. Mannitol symmetry: Mannitol is a symmetrical molecule which can lead to a C1 to C6 scrambling.

Note that due to the differences in the dynamics of the intermediates, these alternative routes contribute on different time scales. For example, the enrichment of the large mannitol pool is very slow, thus the feed of C6 labeled material would be very slow. The flux estimation results described later (Section ’Estimated flux’) show, that FBPase activity (route 2) seems to contribute most (7.25 mmol/Cmol_BM_/h). The route via transaldolase (route 1) and the mannitol metabolism (route 3) were found at lower activities (2.92 and 0.66 mmol/Cmol_BM_/h respectively).

### The C3-C6 fragment of extracellular glucose

For extracellular glucose, a C3-C6 fragment could be measured. According to the labeling composition of the feed, a distribution of 10% m+4, 4% m+1, and 86% m+0 are expected (assuming natural enrichment for the unlabeled carbons of the substrate). However, the measured labeling dynamics not only showed an increase of the fully labeled fraction, but also an m+1 fraction growing steadily up to 11% at the end of the experiment. The enrichment dynamics are comparable to the ones observed in G6P, indicating a metabolic activity leading from intracellular G6P to extracellular glucose.

Alternative routes, e.g. via extracellular trehalose degradation seem less likely because the labeling enrichment is much slower and insufficient to keep the m+1 of extracellular glucose close to m+1 of G6P. To the best of our knowledge, there are no known reports on G6P phosphatase that converts G6P to intracellular glucose, followed by export to extracellular glucose. Nevertheless, due to the strong indications from the data, a G6P phosphatase and a glucose exporter were included in the model to evaluate the hypothesis of a phosphatase activity (Reaction rz_9 and rz_9b in Additional file 3: Table S4).

### The dynamics of pyruvate

The enrichment of pyruvate is much slower compared to its immediate precursor PEP. It is commonly agreed, that alanine transaminase can slow down the labeling. Alanine is a large pool and could additionally be influenced by protein degradation activities. However, the data (Figure 1) clearly indicates that this exchange cannot completely explain the reduced pyruvate enrichment: The enrichment of alanine is actually much faster than pyruvate. Alanine is synthesized in the cytosol, the observed slow enrichment could be a consequence of compartmentation - it seems that mitochondrial pyruvate is enriched much slower than the cytosolic one, the measured combined pool has a slow enrichment profile.

However, all the measured metabolites connected to pyruvate (including amino acids) showed a much higher enrichment than pyruvate, which points to an unlabeled pyruvate inflow to mitochondrial pyruvate. To test this hypothesis, an unlabeled flux producing mitochondrial pyruvate was introduced together with a pyruvate efflux to keep the carbon balance (Reaction rz_8a and rz_8b in Additional file 3: Table S4, rz_8a= rz_8b).

### The dynamics of succinate

It can be assumed that under the studied conditions, succinate is mainly produced via the oxidative branch of the TCA cycle and/or the glyoxylate shunt. In case of the oxidative branch, two of the four carbons of succinate originate from oxaloacetate, the other two from AcCoA (via citrate, isocitrate, alpha-ketoglutarate). The same for the glyoxylate cycle. Two carbons originate from AcCoA (via citrate and isocitrate). As seen in Figure 1 and Additional file 2: Table S6, the m+2 fraction of aspartate and malate are slowly increasing. Oxaloacetate and the two-carbon fragment contributed to succinate are expected to have similar slow labeling dynamics. However, a large fraction of AcCoA originates from fully labeled ethanol. Thus, it is expected that the m+2 fraction of succinate increases immediately after switching to the labeled feed. However, the observed dynamics of m+2 succinate are much slower and only reach about 10% after 1h of labeling. This indicates an additional source of unlabeled AcCoA. The labeling dynamics of leucine (see Additional file 2: Table S6) further supports that there is such an unlabeled source. Therefore, an unlabeled influx into the cytosolic AcCoA pool was introduced into our model (Reaction rz_8d1 and rz_8d2 with equal in- and outflow, Additional file 3: Table S4).

### Dynamics of the unlabeled fractions

**Figure 2:**
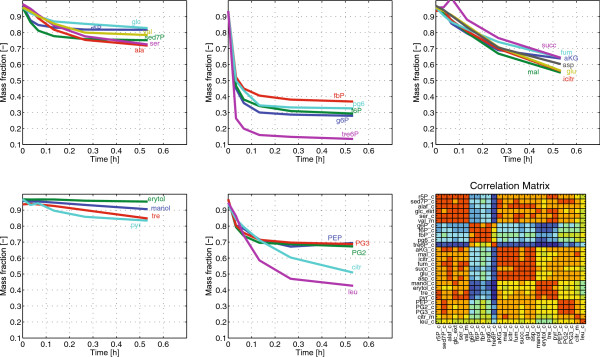
**k-means clustering based on the m+0 time series data.** The first row from left to right are clusters 1 to 3. On the second row from left to right are cluster 4,5, and the color coded correlation matrix (based on m+0 time series correlation).

Besides the oxidative PPP, there are only a few reactions that can lead to unlabeled fractions from the feed labeling. Therefore, the m+0 dynamics is an indication for pool turnover (m+0 is washed out). Figure 2 indicates the similarities of the m+0 labeling dynamics based on k-means clustering in 5 groups. Cluster 2 is the fastest one containing metabolites of upper glycolysis. On the other extreme, the slowest cluster 4 contains storage carbohydrates and pyruvate.

To summarize, the observed labeling dynamics largely support the initial metabolic model (see Materials and Methods), but several extensions are required (see Additional file 3: Table S4):

1. Include trehalose, glycogen, mannitol and further storage pools with respective synthesis and degradation reactions,

2. FBPase activity,

3. A glucose ↔ G6P substrate cycle, including glucose export (Reaction rz_9 and rz_9b);

4. An unlabeled influx into mitochondrial pyruvate (Reaction rz_8a and rz_8b);

5. An unlabeled influx into cytosolic AcCoA (Reaction rz_8d1 and rz_8d2).

### ^13^C based flux estimation

Using the ^13^C metabolic model with the discussed extensions, the free fluxes and unknown concentrations were estimated based on the measured intra- and extracellular concentration and labeling data. The complete set of estimated fluxes and concentrations can be found in Additional file 1: Table S5 and Additional file 1: Table S7. Most of the measured mass isotopomer distributions can be reproduced well (see Figure 1), especially for glycolytic metabolites, pentose phosphate pathway and amino acids. Some TCA cycle metabolites, e.g. succinate, fumarate and malate show some deviations in the later phase.

Most net flux estimations are comparable to the results obtained by MFA. However, several of the fluxes added to the model carry significant flux (Additional file 1: Table S5) and are essential to reproduce the measured labeling enrichments. For the model without the extensions discussed earlier the best fit obtained had a sum of squares of: 12770. Including the additional reactions, the sum of squares is reduced to 1378.4. The F-value becomes F = 51, which is significant. Nevertheless, a chi-square test for the fit of the extended model fails (1378.4 > 810) which is observed more frequently for labeling experiments [25,26].

The sum of ATP consumption due to substrate cycle fluxes is estimated at 42.8 mmol/Cmol_BM_/h. This is 52.1% of the ATP balance gap calculated from metabolic flux analysis. From Table 2, it can be seen that three of the substrate cycles are responsible for the majority of ATP consumption: glycogen synthesis and degradation, G6P-glucose cycling, and FBP-F6P conversions. The contributions from other substrate cycles (trehalose, mannitol, etc.) are much lower.

**Table 2:** **Substrate cycle fluxes at steady-state estimated from instationary**^**13**^**C metabolic flux analysis**

**Name**	**Reaction**	**flux [mmol/Cmol**_**BM**_**/h]**
r3.2	FBPase	7.25 ± 0.91
rz.2b	Trehalose	0.065 ± 0.014
rz.4a+ rz.4a2	Mannitol cycle	1.326 ± 0.162
rz.5a	Erythritol cycle	0.021 ± 0.017
rz.9	g6p phosphatase	10.6 ± 2.1
rz.9c	Polysaccharide degradation	6.47 ± 0.76
rz.8a	Pyr:m recycling	13.1 ± n.d.^*^
rz.8d1	AcCoA recycling	11.3 ± n.d.^*^

^*^The flux standard deviation could not be determined (very high correlation) and was therefore taken out of the calculation. Further parameters/fluxes that were taken out during the statistical calculation are marked in Additional file 1: Table S5 with an asterix.

Additionally, several exchange fluxes were estimated high: e.g. PGI (r1_2), FBP aldolase (r1_4), TPI (r1_4b). These results are consistent with the expectation that PGI and TPI are the most active bidirectional reactions, while others have a lower exchange.

### Enzyme assay

To further support the findings of the ^13^C flux analysis, the enzyme activities of FBPase, PEPCK, and ICL in the cell extract were measured. The FBPase maximal activity was 24 mmol/Cmol_BM_/h. This is 3 times higher than the estimated flux from ^13^C flux analysis (7.25 mmol/Cmol_BM_/h, Table 2). The activities of PEPCK and ICL were much lower (0.70 resp. 10.4 mmol/Cmol_BM_/h).

In an early study on glucose-ethanol mixed substrate cultures of *S. cerevisiae*, Vanrolleghem *et al*. [27] demonstrated the sequential activation of gluconeogenic enzymes in response to an elevated ethanol concentration in the substrate mixture. However, from the enzyme assay results and the estimated fluxes (Table 2), a high FBPase level (r3_2) is observed in *P. chrysogenum* without significant activation of PEPCK (r3_1) and glyoxylate cycle (r5_4).

The activity of extracellular trehalase was only estimated qualitatively by measuring the conversion of trehalose to glucose in broth-filtrate (data not shown). This indicates an extracellular trehalase activity, besides the known membrane-bound ATH1 ortholog.

## Discussion

### Substrate cycles

**Figure 3:**
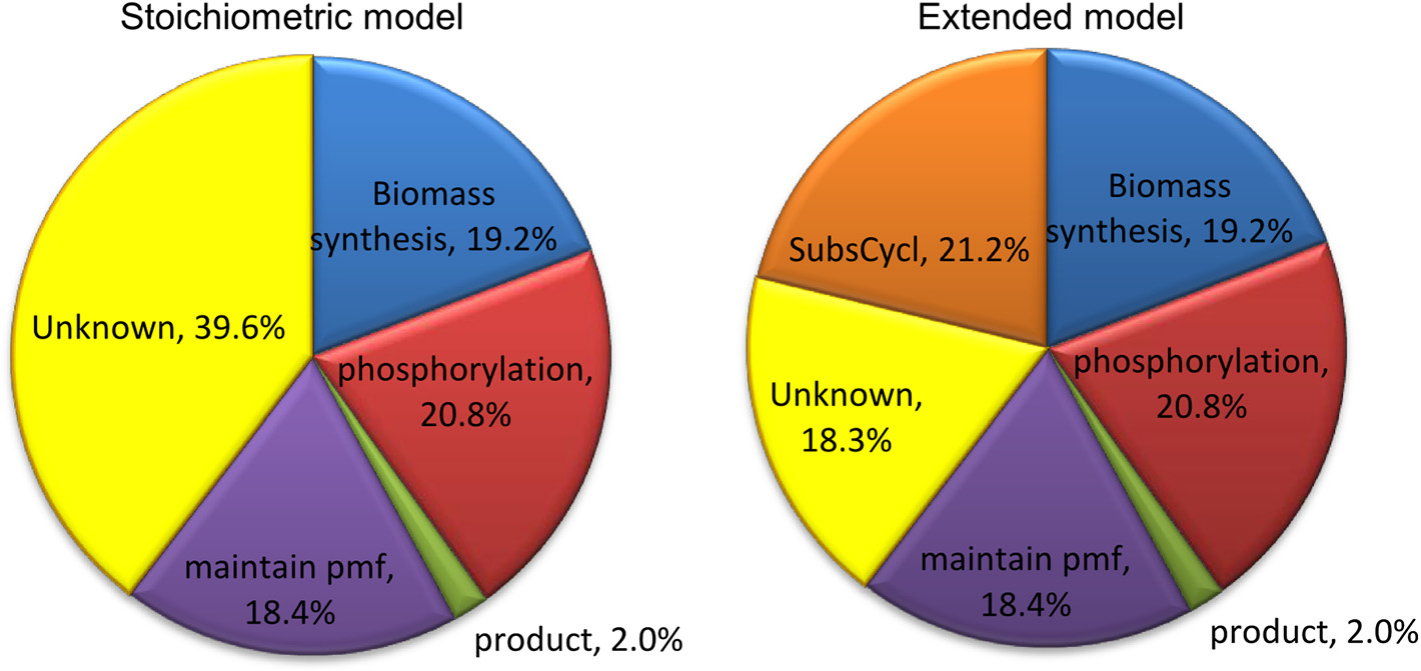
**Comparison of grouped ATP consuming reactions.** Left: based on the results of a classical MFA approach with ATP sink. Right: based on the results from ^13^C flux analysis including substrate cycles.

Based on a detailed INST ^13^C flux analysis, eight cyclic pathways with influence on the ATP balance have been quantified. More than 50% of the energy gap could be attributed to these activities (Figure 3). As shown in Figure 1, extending the metabolic network with substrate cycle fluxes substantially improved the reproduction of the measured labeling data (dashed vs. continuous line).

Most surprisingly, a phosphatase activity, dephosphorylating G6P and an exporter of glucose, showed a high activity. The estimated activity of this cycle strongly depends on the labeling data of extracellular glucose. The labeling has been validated by repeated measurements and samples from non-labeled experiments, confirming the fragment and observed dynamics. Nevertheless, parameter estimation has been performed also without the glucose labeling data to evaluate the impact of these measurements. The intracellular flux distribution did not change significantly, but the dephosphorylation of G6P is estimated lower, at a rate of 3.6 mmol/Cmol_BM_/h. The extracellular sampling protocol has been optimized (cold beads, filtration, liquid nitrogen, lyophilisation) to minimize extracellular conversions after filtration, e.g. trehalose degradation. Therefore it is assumed that no artifacts are measured. The inclusion of a phosphatase reaction does not influence any other fluxes resp. measurements, thus, the estimation relies only on the measured extracellular enrichment data.

A second ATP consuming cycle arises from FBPase activity (7.25 mmol/Cmol_BM_/h, Table 2). This finding could further be supported by the high measured maximal enzyme activity. The recycling of storage metabolites was lower than expected from previous experiments; glycogen and mannitol are found to be the pools with the highest turnover (6.47, 1.33 mmol/Cmol_BM_/h respectively) under steady-state conditions.

To our knowledge, this is the first time that eight different substrate cycle fluxes in an industrial producing strain were systematically quantified using a large scale INST model. The theoretical and experimental approach developed in this study is demonstrated to be a powerful tool to study a large number of parallel/cyclic fluxes in short-term experiments (1 hour of labeling). This approach enables the identification of yet unknown metabolic activities from careful data analysis and the quantification of internal cycles as well as transport cycles.

The total substrate cycle fluxes with the known ATP stoichiometries constitutes half of the estimated ATP consumption in yet unknown reactions (Figure 3). The remaining ATP gap could be a result from a number of other ATP-consuming biological activities that were not accounted for in our model. These activities include unknown transport reactions, protein recycling, DNA repair, and other pathways such as the γ-glutamyl cycle. These activities were not included in our model mainly due to measurement limitations.

But, these reactions have an impact on the labeling dynamics. One example is the mitochondrial pyruvate pool. Here an unlabeled carbon inflow (rz_8a, estimated to be 13.1 mmol/Cmol_BM_/h) was required to reproduce the labeling data. Part of this flux could e.g. be related to pyruvate / valine cycling. Valine is degraded via oxidation to propionyl-CoA and then further converted into succinyl-CoA [28]. Alternatively, the 2-methylcitrate cycle [29], which converts propionyl-CoA into pyruvate could be used. These genes can be found in the genome of *P. chrysogenum*[30,31].

Another reaction that potentially represents an additional substrate cycle is based on the required unlabeled carbon inflow into AcCoA (rz_8d1, estimated to be 13.1 mmol/Cmol_BM_/h). This flux could be a result of fatty acid degradation and/or recycling of amino acids (via glutarate) such as lysine, arginine, valine and α-aminoadipate of which some are intermediates of the penicillin biosynthesis pathway. This could explain the high consumption of ATP associated with the penicillin production (73±20 mol ATP/mol penicillin estimated by van Gulik *et al*. [22]).

### Population heterogeneity

The intracellular concentrations, ^13^C labeling enrichment and extracellular fluxes are based on whole cell population samples. Some recent works demonstrate that substrate cycles can also be associated with population heterogeneity. For example, Aguilar-Osorio *et al.*[32] showed in *Aspergillus niger* that the mannitol synthesis and degrading are active in different cells (vegetative hyphae resp. conidiospores).

Population heterogeneity can also result from stochastic variations of e.g. transcription factors [33]. Rühl *et al*. [34] used the ^13^C enrichment from a specific reporter protein to quantify fluxes of a subpopulation (expressing the protein) in mixed culture.

Besides cell-cycle and stochasticity also the intracellular volume distribution is inhomogeneous between cells. For our calculations, the cell compartment volume distribution and metabolite distribution were assumed to be constant for the whole population. Because the system operates at steady-state (chemostat), there is no time dependent variability in cell size. Therefore the assumption of constant volume distribution only has a limited influence on the results.

### Isotopic dynamics

Due to the presence of large buffering pools with high substrate cycling the isotopic dynamics of the eukaryotic *P. chrysogenum* are slower compared to prokaryotes. Noack *et al*. [35] showed that the upper glycolytic intermediates reach isotopic steady state within 40 seconds at maximal growth rate in *Corynebacterium glutamicum*. The authors also demonstrated that the gap between the short and the long term isotopic steady is very small for glycolytic intermediates, except pyruvate.

## Conclusion

Using ^13^C INST labeling experiments, intracellular fluxes can be determined, including a large set of intracellular and transport cycles. The modeling process led to several additions compared to classical stoichiometric models, e.g. storage metabolism and transport cycles. Our results show, that for filamentous fungi such as *P. chrysogenum*, it is important to take the exchange with the extracellular metabolites into account. For most metabolites an IC/EC ratio of 51 was found, much higher values were obtained for glucose, mannitol, and trehalose. From the extra- and intracellular enrichment, the transport activities could be estimated.

The estimated fluxes were partly supported by enzymatic activity assays. The flux and activity results confirmed a number of observations and hypothesizes from previous studies. These include the derepression of gluconeogenesis under carbon-limited conditions, mobilization of storage carbohydrates and polyols, and the equilibrium state for a number of metabolic reactions. Finally, the ATP-cost of the substrate cycles accounted for 52.1% of the missing ATP in MFA. These give additional insights on potential targets for further improving the yields of substrate.

## Methods

### Strain

A high-yielding *P. chrysogenum* strain (DS17690) was kindly provided by DSM Anti-Infectives (Delft, the Netherlands).

### Design of the labeling experiment

The design of the labeling experiment especially the labeling pattern of the substrate was based on the approach proposed by Nöh *et al*. [36]. The following labeling composition was calculated as most informative and used as substrate feed: 90% [1-^13^C_1_ glucose, 10% [U-^13^C_6_ glucose; 100% [U-^13^C_2_ ethanol. The 10% fully labeled glucose in the substrate mixture improve the information content about the pool turnover of fragment measurements. Samples were taken at 6 different time points (2, 4, 8, 16, 32, 64 minutes) after switching to the labeled medium.

### Medium

Chemically defined medium was used for the chemostat cultivation. Salt and trace element solutions were identical to the ones used by Zhao *et al*. [21]. A mixture of glucose (12.8 g/L) and ethanol (1.7 g/L) was used as carbon source. In the labeled medium, the molar concentrations of glucose and ethanol were identical to the ones of the unlabeled medium. The labeled material was purchased from Sigma (99% atom purity, Cambridge Isotope Laboratories Inc., MA, USA).

### Chemostat cultivation

The chemostat was maintained at a dilution rate of 0.05 h^-1^ in a 1 L bioreactor (Applikon®, Scheidam, the Netherlands). Throughout the experiment, the working volume was kept constant (600 mL) using a level sensor. Basildon® (Abingdon, United Kingdom) antifoam was added during the continuous phase manually with a peristaltic pump (once per day). The airflow into the bioreactor was set to 0.67 vvm. The pH was maintained at 6.5 by addition of 2M NaOH solution, using the Satorious® (Aubagne, France) Biostat B+ controller. The temperature was controlled at 25°C. Biomass dry weight and offgas O_2_ and CO_2_ were monitored using the same methods as in Zhao *et al*. [21].

### The labeling experiment

After five residence times of continuous cultivation, the feed was switched to the labeled medium. The labeled feed was used for 64 minutes, then the feed was switched back to an unlabeled medium until the end of the experiment. The oxygen consumption rate, base addition and regular biomass measurements (data not shown) indicated that the metabolic steady state was maintained throughout the experiment (labeling and back switch). The labeling of the offgas CO_2_ was recorded every minute, using a gas mass spectrometer (Omnistar™ GSD 301, Pfeiffer Vacuum, Germany).

### Intracellular metabolite measurements

Samples for metabolite concentration measurements were taken one hour before the labeling switch using the rapid sampling method of Nasution *et al*. [37]. Internal standard produced from cell extract (*P. chrysogenum*) grown on fully ^13^C labeled medium was used to improve the quantification accuracy [38,39].

### Sample and data processing

All quenched/washed biomass samples were immediately extracted using boiling ethanol [37,40]. Mass isotopomer samples were analyzed by LC/MS [14] and GC/MS [40,41]. For the analysis with GC-MS, 100 μL of sample are freeze-dried and then derivatized with 50 μL pyridine (HPLC grade 99.9%, Sigma-Aldrich, Buchs, Switzer-land) containing 20 g/L O-Methoxyamine-hydrocloride (MOX, purum, Sigma-Aldrich, Buchs, Switzerland) for 50 min at 70°C. Then 80 μL of N-methyl-N-trimethylsilyltrifuoroacteamide (MSTFA, Thermo scientific, Bellafonte, PA, USA) are added and incubated at 70°C for another 50 min. Details of the injection and oven program can be found in [41].

The mass shifts of unlabeled samples compared to ^13^C-labeled cell extract were used to confirm the metabolite fragments (data not shown). To check for possible interfering compounds, the mass istotopomer distribution of cell extract from an unlabeled culture was measured and confirmed by the expected spectra (natural labeling distribution).

The measured peak area data was then processed using the mass correction tool [42] to correct for the influence of derivatization agents and non-carbon atoms. Intracellular metabolite concentrations were calculated based on the IDMS method of Mashego *et al*. [38] and Wu *et al*. [39]. A cellular volume of 2.5 mL/gDW [43] was assumed to calculate the intra- to extracellular concentration ratios.

For extracellular measurement, 2mL of broth were withdrawn and collected in a syringe packed with cold beads (−20°C) leading to a mixing temperature of about 4°C. The cooled broth was filtrated as fast as possible. Then, 20 μL were transferred to a GC glass vial and immediately placed in liquid nitrogen and freeze dried. The dried sample was stored at −80°C until analysis.

### Enzyme assays

Samples from independent chemostats under identical condition were used for enzyme assays [44]. Enzyme activities of PEP carboxykinase (PEPCK) and FBPase were analyzed using adapted protocols of Harris *et al*. [45]. A conversion factor for the measured enzyme activity (1 μmol/(mg protein)/min equals 417 mmol/Cmol_BM_/h) was used to compare the enzyme activity with the metabolic fluxes.

### Metabolic flux analysis

Metabolic flux analysis using an adapted model of van Gulik *et al*. [23] was performed as a reference calculation. The reactions in this model are listed in Additional file 4: Table S2.

The metabolic network for ^13^C flux analysis was derived from this stoichiometric model. All extensions concerning substrate cycles are explained in the following sections. Further differences, especially lumping of biomass reactions are explained in detail in the supplement and listed in Additional file 2: Table S4. The metabolic model used in this study contains 94 metabolites and 160 metabolic reactions (resp. 196 when including the 36 backward fluxes of bidirectional reactions). Of these, 37 fluxes are biomass synthesis reactions which were constrained based on the measured biomass growth rate and the biomass composition. Seven reaction rates were constraint based on assumptions (like non-active succ/fum shuttle (rz7c, forward and backward), non-active rz7 (forward and backward), in- and outflow of yet unknown reactions into pyruvate and AcCoA (rz_8a=rz_8b and rz_8d1= rz_8d2), and the value for r2.2 was taken from the MFA approach). Additionally five fluxes describing the labeling inflow (rLUGlc, rL1Glc, rLNGlc, rLUEth, rLNEth) were set to the measured rates and used labeling mixture. Furthermore, it was assumed that reactions producing a symmetrical molecule (like mannitol, succinate and fumerate) do produce the two possible species equally – leading to another three constraints.

The flux estimation was performed based on the cumomer concept described by [46] which can be adapted to isotopic dynamic conditions as described by Nöh *et al*. [16]. The numeric simulation and parameter estimation was performed using the software gPROMS (PSE, London, UK).

The free fluxes and unmeasured concentrations were estimated by minimizing the deviations between simulated and measured time series of mass isotopomer distributions (Additional file 2: Table S6). The optimization algorithm SRQPD (an adapted sequential quadratic programming method) implemented in gPROMS was used [47]. A total of 800 mass fractions were measured from 28 metabolites collected at 6 different time points (listed in Additional file 2: Table S6), as well as 26 measured metabolite concentrations. Fifty free fluxes and nine concentrations were estimated, including eight different substrate cycle fluxes. For the statistical evaluation a series of fluxes was taken out (fixed), these are labeled (*) in Additional file 1: Table S5.

### Metabolic model and biomass formation reactions

Modeling of the ^13^C distributions requires detailed knowledge about the metabolic networks and the atom transitions of all enzymatic reactions [46]. A metabolic network model of *P. chrysogenum* was first developed by Jorgensen *et al*. [48] and extended by van Gulik *et al.*[23]. These models were later adapted for the use in ^13^C flux analysis based on isotopic steady state ^13^C distributions of the proteinogenic amino acids [25,49,50]. All these isotopic steady state models assume a unidirectional biomass formation, including the synthesis of protein, nucleotides, lipids, and carbohydrates, which originate from their respective precursors in the central carbon metabolism.

Recently, increasing attention was drawn to the reversibility of anabolic reactions. Grotkjaer *et al*. [51] discussed the impact of bidirectional transaminase activities and protein turnover on the labeling dynamics. Supporting evidence for protein turnover in *E. coli* was found by Shaikh *et al*. [52] using inducible green fluorescent protein (GFP). Also for storage carbohydrate pools, evidence of constant turnover has been found, especially by ^13^C off gas CO _2_labeling data in *S. cerevisiae*[53].

Although these cyclic fluxes result in considerable ATP consumption, few ^13^C flux analysis studies have taken the bidirectionality of these fluxes into account [54-56]. Furthermore, most studies do not consider substrate cycles in central carbon metabolism based on the applied experimental conditions. In the following sections a brief overview of possible substrate cycles and the related pools will be given to describe the metabolic model for INST^13^C flux analysis.

### Metabolism of mannitol and other polyols

**Figure 4:**
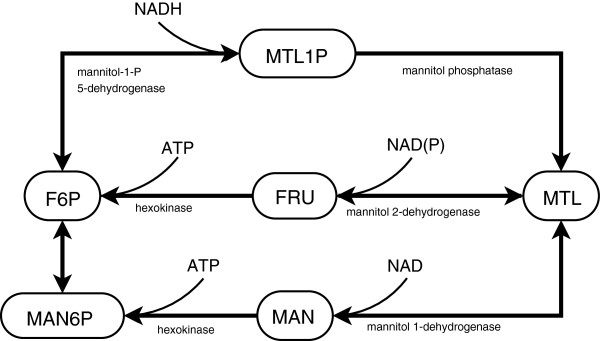
Reaction scheme representing the reported mannitol synthesis and degradation reactions.

**Figure 5:**
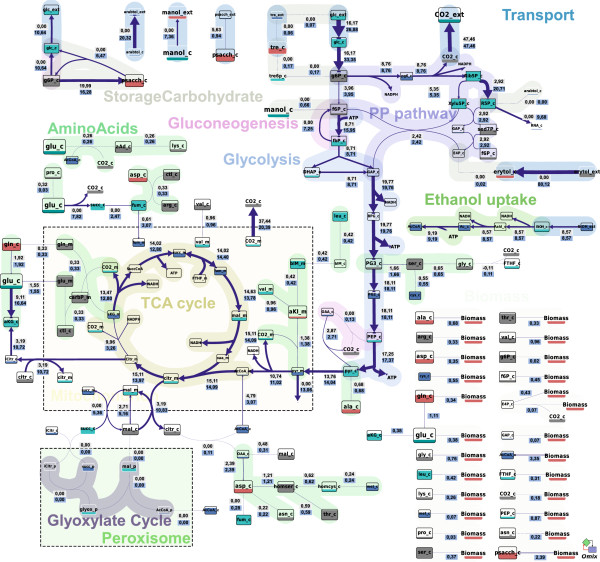
**Metabolic flux distribution based on ^13^C flux analysis using the extended metabolic model (on blue) in comparison to the result obtained from classical MFA (on grey).** The arrow sizes correspond to the net flux values shown on blue background in mmol/Cmol_BM_/h. Metabolites are categorized into 5 concentration ranges using different colors and sizes. Within each concentration range, the fill-level of the square further shows the relative concentration to the reference concentration of the corresponding range. Fully filled block of light turquoise, grey, red, and blue color corresponds to metabolite concentration of 0.13, 0.78, 4.8, and 44 mmol/Cmol_BM_. Image prepared using the visualization software Omix [61].

Mannitol is usually the most abundant soluble carbohydrate in the mycelia of fungi [57]. It is considered to play various physiological roles: carbohydrate storage, overflow reservoir for reducing power, but also functions as stress protectant [58] (and references therein). Despite of its metabolic significance, the details of the synthesis and degradation pathways partly remain unknown [58]. There seem to be three alternative routes (Figure 4) between F6P and mannitol. These routes can be discriminated by their unique intermediates: mannitol-1-phosphate (MTL1P), fructose (FRU), and mannose (MAN). In filamentous fungi, it was shown that mannitol is synthesized via MTL1P in the vegetative hyphae, and degraded via fructose in the conidiospores [32]. The cofactor (NADH/ NADPH) specificity of mannitol 2-dehydrogenase is still unknown; Nevertheless, it is clear that a cycle of production and degradation will require ATP [58] (Figure 5).

In ^13^C flux analysis it has to be assumed that the culture is homogeneous – currently neither subpopulation sampling nor cell cycle synchronization protocols are available. For the whole cell population, a mixture of differentiated hyphal cells and conidiospores results in a bidirectionality of the reaction between F6P and mannitol. In the studied condition, the concentrations of MTL1P and FRU were negligible compared to mannitol and these pools were lumped with the intracellular mannitol pool.

Mannitol, erythritol, and arabitol were detected in both the mycelia and the supernatant. Therefore, our model also included the synthesis and degradation pathways between the phosphorylated sugar, and their corresponding C4, C5, and C6 sugar alcohols, as well as secretion and uptake fluxes (see Additional file 3: Table S4).

### Trehalose metabolism

Simultaneous synthesis and degradation of trehalose combined with hexokinase activity results in an ATP-consuming metabolic cycle. For the degradation pathway in fungi, two different trehalases are reported [59]. In *S. cerevisiae*, acid trehalase is known to be targeted at the cell surface [60,61] and responsible for extracellular degradation of trehalose (in *S. cerevisiae*, ATH1) [62]. One ortholog of ATH1 was found in the genome of *P. chrysogenum* (Pc16g11870) [30]. Neutral trehalase is responsible for the cytosolic activity in *S. cerevisiae* (NTH1) [59]. An ortholog was found in *P. chrysogenum* (Pc22g03670 [30]). Because trehalose is also found in the extracellular filtrate (see section Results), its secretion and extracellular degradation were included in our metabolic model.

### Glycogen

Glycogen can be hydrolyzed into G6P via glycogen phosphorylase (Pc13g11660) or glucose via glucan 1,4-alpha-glucosidase (Pc16g00620 and Pc13g11940) [30,44]. Unfortunately, the labeling enrichment of glycogen could not be measured as no appropriate (specific) protocol was available. Therefore, glycogen was only included as a simulated pool.

### Central carbon metabolism

Gluconeogenesis reactions and anaplerotic reactions can lead to ATP-consuming cycles. In our model, the cycles between GLC-G6P, F6P-FBP, PEP-PYR-OAA are considered. Furthermore, pyruvate carboxylase, glyoxylate shunt, amino acid biosynthesis. Transaminases using glutamate, aspartate, and alanine are also included. To reduce the model size, the β-lactam synthesis and the three large aromatic amino acids: phenylalanine, tyrosine, and tryptophan, were described by their precursors. The pathways of glycolysis, pentose phosphate pathway (PPP), tricarboxylic acid (TCA) cycle were modeled the same as in van Gulik *et al*. [22]. In addition, glutamate degradation to succinate as well as a cytosolic fumarate reductase were added to the model based on the high expression level of the corresponding genes [44] in previous studies under similar conditions. Moreover, degradation of RNA was included in the model (reaction rz6c in Additional file 3: Table S4).

All the mentioned exchanged fluxes were still not sufficient to explain the slow labeling enrichment of AcCoA and pyruvate. Therefore, additional unlabeled inputs were added to these pools and estimated during parameter optimization using a similar approach as van Winden *et al*. [14]. These unlabeled inputs could result from fatty acid degradation and amino acid degradation. All the reactions and the atomic transitions used in our model are listed in Additional file 3: Table S4.

### Metabolite concentration

The isotopomer distribution dynamics do not only depend on the fluxes but also the metabolite concentrations. Most metabolite concentrations could be measured by LC-MS or GC-MS (see Table 1). For metabolites present in different compartments, the following assumptions were used:

1. the mitochondria and the peroxisome occupy 10% of the total cellular volume [1];

2. there are no significant concentration gradients between different compartments.

Metabolite concentrations that could not be measured were estimated by parameter estimation (as additional parameter).

## Endnotes

^a^A metabolite present in cytosol and mitochondria is distributed 10% mitochondria, 90% cytosol, if additionally present in the peroxisome the distribution becomes 10% mitochondria, 10% peroxisome, 80% cytosol.

## Abbreviations

AAD: α-Aminoadipic acid; Ac: Acetate; AcCoA: Acetyl-CoA; AKG: α-Ketoglutarate; AKI: α-Keto-isovalerate; ALA: Alanine; ARA: Arabitol; ARG: Arginine; ASN: Asparagine; ASP: Aspartate; bIM: β-isopropylmalate; BPG: 1,3-bisphosphoglycerate; CarbP: Carbamoyl phosphate; CITR: Citrate; CO2: Carbon dioxide; CTL: Citrulline; CYS: Cysteine; DHAP: Dihydroxyacetonephosphate; E4P: Erythrose 4-Phosphate; Erytol: Erytritol; ESEA: Ergosterolester (and precursors); EtOH: Ethanol; F6P: Fructose-6-Phosphate; FBP: Fructose-1,6,-bis-Phosphate; FTHF: 5-Formyltetrahydrofolate; FUM: Fumarate; G3P: Glycerol-3-Phosphate; G6P: Glucose-6-Phosphate; GAP: Glyceraldehyde 3-Phosphate; GLC: Glucose; GLN: Glutamine; GLU: Glutamate; GLY: Glycine; GLYOX: Glyoxylate; HOMCYS: Homocysteine; HOMSER: Homoserine; ICITR: Isocitrate; ILE: Isoleucine; L1Glc: [1-^13^C] glucose; LEU: Leucine; LNEtOH: Ethanol (natural labeling enrichment); LNGlc: Glucose (natural labeling enrichment); LUEtOH: Uniformly labeled [U-^13^C_2_] ethanol; LUGlc: Uniformly labeled [U-^13^C_6_] glucose; LYS: Lysine; MAL: Malate; Manol MTL: Mannitol; MET: Methionine; MYTHF: Methyltetrahydrofolate; mRNA: Pool containing messenger and other RNAs in the cell (assumed composition: CH_1.23_N_0.416_O_0.715_P_0.104_); OAA: Oxaloacetate; PEP: Phosphoenolpyruvate; PG2 2PG: 2-Phosphoglycerate; PG3 3PG: 3-Phosphoglycerate; PG6: 6-phosphogluconate; PhetaA: Phosphatidylethanolamine (and precursors); PRO: Proline; PSacch: Polysaccharide (like Glycogen); PYR: Pyruvate; R5P: Ribulose-5-Phosphate; Rbtol: Ribitol; Rib5P: Ribose 5-Phosphate; SED7P: Sedoheptulose 7-Phosphate; SER: Serine; SUCC: Succinate; THR: Threonine; TRE: Trehalose; TRE6P: Trehalose-6-Phosphate; VAL: Valine; Xylu5P: Xylulose 5-Phosphate.

## Competing interests

The authors declare that they have no competing interests.

## Authors' contributions

ZZ Performed the experiments, modeling and evaluation of data and wrote the manuscript, AP Performed the GC-MS analysis of all samples, LJ Assisted significantly during the experiments, JJH assisted in the supervision of the study, AW supervised the experiments, modeling and participated in writing of the manuscript.

## Supplementary Material

Additional file 1 **Table S1.** Measured biomass specific uptake and secretion rates [mmol/Cmol/h]. Table S3: Metabolic flux analysis results [mmol/Cmol/h]. Table S5: 13C flux analysis results*. Tabel S7: Measured, assumed and estimated concentrations (intracellular μmol/gDW, extracellular mmol/L). Click here for file

Additional file 2 **Table S6.** Measured mass isotopomer ratios. Click here for file

Additional file 3 **Table S4.** Metabolic reaction network with atom transitions. Click here for file

Additional file 4 **Table S2.** Metabolic network used for the metabolic flux analysis (unlabeled). Click here for file
