# Supplementary material for: Substrate cycles in Penicillium chrysogenum quantified by isotopic non-stationary flux analysis
Source: Microb Cell Fact. 2012 Oct 25;11:140. doi: 10.1186/1475-2859-11-140 (PMC3538697; doi:10.1186/1475-2859-11-140)
Supplement: Additional file 1 — Table S1. Measured biomass specific uptake and secretion rates [mmol/Cmol/h]. Table S3: Metabolic flux analysis results [mmol/Cmol/h]. Table S5: 13C flux analysis results*. Tabel S7: Measured, assumed and estimated concentrations (intracellular μmol/gDW, extracellular mmol/L). [file 1475-2859-11-140-S1.pdf]

Supplement

^{13}C metabolic model

The metabolic model used in the ^{13}C flux analysis was derived from Table S2. Besides the extensions introduced in the second section, several further adaptations were made: The most observable change is the removal of many reactions due to the absence of the corresponding metabolites in the ^{13}C simulation. These metabolites are either without carbon atoms or only conserved moieties. These reactions include: r10.1, r10.2, r10.3, r10.5, r10.9, r10.20, r11.11, r11.12, r12.1, r12.2, r13.1–r13.9, r14.1, and r15.1. α-ketobutarate was also removed because its concentration was below the detection limit. This resulted in the lumping of r11.35, r11.36, and r11.37 into a modified r11.35 in the ^{13}C metabolic model. Similar lumping was also applied to the citruline transport reaction (r10.7), fatty acid synthesis (reactions r16.x), and C1-metabolism (r8.2, r8.3, and r8.5). Biomass formation reactions r18.18 and r18.3. These lumped reactions are named 'bm_*' in the ^{13}C metabolic model. To further reduce the size of the derived isotopomer model, the biosynthesis reactions of the ACV and the aromatic amino acids phenylalanine, tryptophan, and tyrosine were modeled as efflux reactions from the corresponding precursors cysteine, valine, serine, PRPP, PEP, E4P. The penicillin synthesis reactions r19.1, r19.2, r19.3, and r19.4 were also converted to effluxes from the respective precursors. To simulate the labeling of symmetrical metabolites, a pair of identical reactions using opposite atomic transitions were introduced: the reactions r4_7x, r4_7y, r4_9x, r4_9y, rz_4a, and rz_4a2. Finally, to keep compatibility with 13C-FLUX, all reactions in the ^{13}C metabolic model contain no more than two substrates and two products. In case more

than two reactants were present, reactions were split. This resulted in reactions r11_21a, r11_21b, r11_32a, and r11_32b.

Mass isotopomer deconvolution

Fragmentation of metabolites increases the information content about the labeling distribution. Especially, when several fragments are measured, the data can be used to estimate further, non-measured linear combinations of isotopomers. Especially, relations of signals can be used [38, 62]. e.g. for G6P, the complete molecule containing six carbons and a fragment containing the last four carbons (C3-C6) could be measured. The C1-C2 fragment is unmeasured. The distributions of the different fragments are related. e.g. a fully labeled whole fragment would produce two fully labeled fragments, resp. two fully labelled fragments will result in a fully labelled whole molecule. In general, the measurement of the whole molecule can be calculated from the two fragment measurements (W: C1-C6 G6P, V:C3-C6 fragment, U:C1-C2 fragment with the mass isotopomer distributions $\mathbf{w} = \left(w_0, w_1, \ldots w_6\right)^T$, $\mathbf{v} = \left(v_0, v_1, \ldots v_4\right)^T$ and $\mathbf{u} = \left(u_0, u_1, u_2\right)^T$):

$$\underbrace{\begin{matrix} w_0 \\ w_1 \\ w_2 \\ w_3 \\ w_4 \\ w_5 \\ w_6 \end{matrix}}_{\mathbf{w}} \begin{matrix} = \\ = \\ = \\ = \\ = \\ = \\ = \end{matrix} \underbrace{\begin{matrix} u_0\, v_0 \\ u_1\, v_0 + u_0\, v_1 \\ u_2\, v_0 + u_1\, v_1 + u_0\, v_2 \\ u_2\, v_1 + u_1\, v_2 + u_0\, v_3 \\ u_2\, v_2 + u_1\, v_3 + u_0\, v_4 \\ u_2\, v_3 + u_1\, v_4 \\ u_2\, v_4 \end{matrix}}_{\mathbf{B}\left(\mathbf{v}\right)\mathbf{u}} \quad \text{with: } \mathbf{B}(\mathbf{v}) = \begin{pmatrix} v_0 & & \\ v_1 & v_0 & \\ v_2 & v_1 & v_0 \\ v_3 & v_2 & v_1 \\ v_4 & v_3 & v_2 \\ & v_4 & v_3 \\ & & v_4 \end{pmatrix} \qquad (1)$$

The equations can be rewritten to calculate the distribution of the unmeasured C1-C2 (u) fragment as a function of the measurements of C1-C6 (**w**) and C3-C6 (**v**). This system has ‘hidden’ additional equations, e.g. $\sum_u u_i = 1, \sum_v v_i = 1, \sum_w w_i = 1$. Also,

negative fractions are physically impossible. Therefore, the solution for the non-measured fragment U is best obtained by:

$$\hat{\mathbf{u}} = \underset{\substack{\mathbf{u} \geq \mathbf{0} \\ \sum \mathbf{u} = 1}}{\arg\min_{\mathbf{u}}} \left\| \mathbf{w} - \mathbf{B}(\mathbf{v})\mathbf{u} \right\| \qquad (2)$$

The optimization can be solved using quadratic programming, here quadprog of MatLab was used.

Table S1: Measured biomass specific uptake and secretion rates [mmol/Cmol/h]

**Name**	**Rate [mmol/Cmol/h]**		
**biomass**	54.66	±	1.33
**CO2:ext**	47.37	±	2.17
**EtOH:ext**	-8.55	±	0.31
**ExPept:ext**	2.42	±	0.32
**glc:ext**	-16.12	±	0.58
**O2:ext**	-52.44	±	2.13
**PAA:ext**	-0.51	±	0.04
**penG:ext**	0.51	±	0.05
**psacch:ext**	5.66	±	0.76

Table S3: Metabolic flux analysis results [mmol/Cmol/h].

r1.1	16.17	r10.18	0.33	r11.16	2.39	r11.32	0.14	r13.5	0.05	r16.5	0.12	r19.4	0.51	r4.2	15.11	r8.2	0.18
r1.2	3.96	r10.19	0.42	r11.17	0.22	r11.33	0.42	r13.6	0.05	r16.6	0.12	r2.1	8.76	r4.3	15.11	r8.3	0.36
r1.3	8.71	r10.2	4.79	r11.18	1.21	r11.34	0.53	r13.7	-0.06	r16.7	0.11	r2.2	8.76	r4.4	9.96	r8.4	0.16
r1.4	8.71	r10.20	4.79	r11.19	0.59	r11.35	0.55	r13.8	0.16	r16.8	0.09	r2.3	2.92	r4.5	1.97	r8.5	0.16
r1.5	19.77	r10.21	4.79	r11.2	1.92	r11.36	0.55	r13.9	0.05	r16.9	0.09	r2.4	5.35	r4.6	3.19	r9.1	38.19
r1.6	19.77	r10.24	0.55	r11.20	0.24	r11.37	0.55	r14.1	0.30	r17.1	19.99	r2.5	2.92	r4.7	13.47	r9.11	0.51
r1.7	18.11	r10.3	128.90	r11.21	0.26	r11.4	0.33	r15.1	82.09	r17.2	0.45	r2.6	2.92	r4.8	14.02	r9.13	2.41
r1.8	18.11	r10.5	168.40	r11.22	0.68	r11.5	0.33	r16.1	0.02	r17.3	0.14	r2.7	2.42	r4.9	14.02	r9.14	5.63
r1.9	17.25	r10.6	3.19	r11.23	1.38	r11.6	0.33	r16.10	0.05	r17.4	0.14	r21.1	0.51	r5.1	2.87	r9.15	81.58
r10.1	171.10	r10.7	0.33	r11.24	0.96	r11.7	0.26	r16.11	0.02	r17.5	0.05	r21.13	1.02	r5.3	0.48	r9.18	8.57
r10.10	0.26	r10.8	2.71	r11.25	0.42	r11.8	0.26	r16.12	0.01	r17.6	0.05	r21.14	-1.02	r6.1b	49.82	r9.2	0.41
r10.11	13.76	r10.9	0.26	r11.26	0.42	r11.9	1.66	r16.13	0.16	r17.7	0.05	r21.15	1.02	r6.2b	39.00	r9.20	10.69
r10.12	37.44	r11.1	9.11	r11.27	0.43	r12.1	4.86	r16.14	0.03	r17.8	0.07	r21.2	0.51	r6.3b	14.02	r9.3	16.17
r10.13	0.61	r11.10	0.65	r11.28	0.25	r12.2	4.86	r16.15	0.03	r18.18	8.19	r21.6	0.51	r6.4	158.60	r9.4	0.62
r10.14	3.19	r11.11	0.62	r11.29	0.13	r13.1	0.18	r16.16	0.03	r18.3	46.40	r21.7	0.51	r7.1	8.57	r9.6	52.35
r10.15	0.26	r11.12	0.62	r11.3	0.32	r13.2	0.10	r16.2	0.12	r19.1	0.51	r4.1	10.74	r7.2	9.19	r9.7	47.46
r10.16	1.55	r11.13	0.62	r11.30	0.05	r13.3	0.08	r16.3	0.29	r19.2	0.51	r4.10	14.63	r7.3	8.57	r9.8	0.51
r10.17	0.96	r11.14	0.62	r11.31	0.49	r13.4	0.12	r16.4	0.27	r19.3	0.51	r4.11	15.11	r8.1	-0.11		

Table S5: ^{13}C flux analysis results*

intra- and extracellular fluxes (mmol/Cmol/h)											
r1.1F	33.35	r2.6F	12.08	r5.5F	0.00	r11.1B	44.63	r11.24F	0.96	rz.5bB	80.12
r1.2B	186.53	r2.7B	23.76	r7.1F	8.57	r11.1F	61.26	r11.25F	0.42	rz.5bF	80.12
r1.2F	190.48	r2.7F	26.19	r7.2F	9.19	r11.2B	7.88	r11.26F	0.42	rz.6aB	0.00
r1.3F	15.95	r3.1F	0.13	r8.1F	0.11	r11.2F	9.80	r11.32aF	0.14	rz.6aF	0.00
r1.4bB	38.78	r3.2F	7.25	r8.4F	0.16	r11.3F	0.03	r11.32bF	0.14	rz.6bB	20.32
r1.4bF	47.48	r4.1F	11.02	r9.3F	26.88	r11.4F	0.33	r11.35F	0.55	rz.6bF	20.32
r1.4B	14.04	r4.2F	14.09	r9.7F	47.46	r11.5F	0.33	r16.9F	0.09	rz.6cB	9.68
r1.4F	22.74	r4.3B	61.27	r9.14F	0.94	r11.6F	0.33	r16.13F	1.58	rz.6cF	9.68
r1.5B	342.16	r4.3F	75.25	r9.18F	8.57	r11.7F	0.26	r17.1F	16.28	rz.7bB	0.01
r1.5F	361.92	r4.4F	3.26	r10.4B	7.73	r11.8F	0.26	r21.8F	0.00	rz.7bF	2.47
r1.6B	0.35	r4.6F	10.72	r10.4F	13.10	r11.9F	1.66	r21.10F	0.00	rz.7cB*****	0.00
r1.6F	20.12	r4.7xF	2.40	r10.6B	0.57	r11.10F	0.65	r21.11F	0.00	rz.7cF*****	0.00
r1.7B	245.19	r4.7yF	2.40	r10.6F	11.28	r11.13F	0.62	rz.1F	0.17	rz.7B*****	0.00
r1.7F	263.29	r4.9xB	3.43	r10.8F	5.16	r11.15F	0.00	rz.2aF	0.17	rz.7F*****	0.00
r1.8B	134.39	r4.9xF	8.78	r10.10F	0.26	r11.16B	47.86	rz.2bF	0.07	rz.8aF	13.06
r1.8F	152.50	r4.9yB	3.43	r10.11F	14.04	r11.16F	50.25	rz.3bF	0.07	rz.8bF	13.06
r1.9F	17.37	r4.9yF	8.78	r10.12F	20.39	r11.17B	0.00	rz.3F	0.00	rz.8c1F	7.82
r2.1F	8.76	r4.10B	237.79	r10.13F	3.07	r11.17F	0.22	rz.4a2B	0.59	rz.8d1F*****	11.32
r2.2F*	8.76	r4.10F	251.57	r10.14B	0.36	r11.18F	1.21	rz.4a2F	0.66	rz.8d2F*****	11.32
r2.3B	17.78	r4.11B	0.10	r10.14F	11.19	r11.19F	0.59	rz.4aB	0.59	rz.9bF	10.64
r2.3F	20.71	r4.11F	14.19	r10.15F	0.26	r11.20F	0.24	rz.4aF	0.66	rz.9c2F	6.47
r2.4B	95.00	r5.1F	2.71	r10.16F	1.55	r11.21aF	0.26	rz.4bB	7.36	rz.9cF	6.47
r2.4F	100.35	r5.2F	0.11	r10.17F	0.96	r11.21bF	0.26	rz.4bF	7.36	rz.9dF	0.10
r2.5B	0.00	r5.3B	0.30	r10.18F	0.33	r11.22B	15.24	rz.4cF	0.14	rz.9F	10.64

r2.5F	2.92	r5.3F	0.61	r10.19F	0.42	r11.22F	15.91	rz.5aB	0.02		
r2.6B	9.16	r5.4F	0.00	r10.21F	3.07	r11.23F	1.38	rz.5aF	0.02		

biomass synthesis fluxes (mmol/Cmol/h)

bm_accoa*	3.35	bm_aspfum*	0.28	bm_eseA*	1.58	bm_glnglu*	1.11	bm_lys*	0.26	bm_pro*	0.03
bm_akg*	0.38	bm_co2*	0.18	bm_f6p*	0.45	bm_glu*	0.38	bm_met*	0.07	bm_prpp*	0.35
bm_ala*	0.68	bm_cys*	0.55	bm_fthf*	0.31	bm_gly*	0.76	bm_myth*f	0.16	bm_psacch*	2.39
bm_arg*	0.33	bm_e4p*	0.43	bm_g6p*	0.02	bm_his*	0.14	bm_pep*	0.87	bm_pyr*	0.05
bm_asn*	0.22	bm_e4p0*	0.07	bm_gap*	0.07	bm_ile*	0.26	bm_pep2*	0.81	bm_ser*	0.37
bm_asp*	0.35	bm_e4p1*	0.43	bm_gln*	0.34	bm_leu*	0.42	bm_phetaA*	0.09	bm_thr*	0.33
										bm_val*	0.96

Flux names ending on ’F’ are forward fluxes. ’B’ represent backward fluxes. Fluxes with an asterisk ‘’ have been excluded from the statistical analysis (fixed). The biomass synthesis fluxes are calculated based on the biomass composition and the growth rate.

Tabel S7: Measured, assumed and estimated concentrations (intracellular µmol/g_{DW}, extracellular mmol/L).

Met.	c	Met.	c	Met.	c	Met.	c	Met.	c	Met.	c	Met.	c	Met.	c
aAd_c*	2.16	bIM_m+	0.61	E4Pbio*	3.61	G6P_c	3.29	HOMCYS_c	0.18	MAL_p	0.25	PG6_c	0.07	SER_c	4.77
AcCoA_c*	0.10	BPG_c*	0.004	E4Pbio2*	3.61	GAP_c+	0.01	HOMSER_c	3.61	Manol_c	174.73	PhetaA_c*	3.61	SUCC_c	0.50
AcCoA_m*	0.01	carbP_m*	3.61	Erytol_c	9.21	GLC_c	0.65	ICITR_c	0.033	Manol_ext	0.58	PRO_c	0.78	SUCC_m	0.06
AcCoA_p*	0.01	CITR_c	1.53	Erytol_ext	0.12	GLC_ext	0.002	ICITR_m	0.004	MET_c	0.08	Psacch_c+	22.47	SUCC_p	0.06
aKG_c	0.52	CITR_m	0.15	ESEA_c*	3.61	GLN_c	26.32	ICITR_p	0.004	mRNA+	539	PYR_c	0.46	THR_c	4.33
aKG_m	0.06	CO2_c*	1.30	EtOH_c*	0.18	GLN_m	2.63	ILE_c	0.24	MYTHF_c*	0.18	PYR_ext	0.04	THR_m	0.04
aKI_m+	5.60	CO2_ext+	11.5	EtOH_ext	0.67	GLU_c	44.40	ILE_m	0.02	OAA_c+	0.04	PYR_m	0.05	TRE_c	20.40
ALA_c	13.75	CO2_m	0.14	F6P_c	0.68	GLU_m	4.44	ILEA_m*	3.61	OAA_m+	0.14	R5P_c*	0.74	TRE_ext	0.02
ARG_c	3.61	ctl_c*	3.61	FBP_c	0.36	GLY_c	1.48	LEU_c	0.69	PEP_c	0.16	Rbtol_c	4.01	TRE6P_c	0.20
ASN_c	1.23	CYS_c	0.12	FTHF_c*	0.18	GLYOX_p*	0.18	LYS_c	0.99	PEPbio*	3.61	Rbtol_ext	0.03	VAL_c	2.11
ASP_c	14.69	DHAP_c	0.13	FUM_c	0.70	HIS_c	1.05	MAL_c	1.99	PG2_c	0.08	Rib5P_c	0.72	VAL_m	0.23
bIM_c+	0.0001	E4P_c	0.004	FUM_m	0.08	HISA_c*	3.61	MAL_m	0.25	PG3_c	0.87	Sed7P_c	2.20	Xylu5P	0.74

All concentrations in µmol/g_{DW}, except extracellular metabolites ('_ext') in mmol/L.

* fixed value (assumption). Based on preliminary results (AcCoA), thermodynamic considerations (R5P (eq. with Xylu5P), EtOH (eq. with extracellular space) or when unknown set to 0.1 mmol/Cmol (3.61 µmol/g_{DW}) except BPG (0.0001 mmol/Cmol).

+ determined from parameter estimation. The initial value for CO2_ext in the extracellular space is based on calculations (Henry's law and bicarbonate equilibrium), resulting in a pool concentration of 0.54 mmol/L. The large deviation could originate from the headspace and delays in the MS device. The initial value for the mRNA pool size originates from the biomass composition (431 µmol/g_{DW}).
