# Supplementary material for: Substrate cycles in Penicillium chrysogenum quantified by isotopic non-stationary flux analysis
Source: Microb Cell Fact. 2012 Oct 25;11:140. doi: 10.1186/1475-2859-11-140 (PMC3538697; doi:10.1186/1475-2859-11-140)
Supplement: Additional file 2 — Table S6. Measured mass isotopomer ratios. [file 1475-2859-11-140-S2.docx]

**Table S2**: Metabolic network used for the metabolic flux analysis (unlabeled).

| ID | Reaction |
| --- | --- |
| R1.1 | 1 ATP:CYT + 1 GLC:CYT ⇒ 1 ADP:CYT + 1 G6P:CYT + 1 H:CYT |
| R1.2 | 1 G6P:CYT ⇒ 1 F6P:CYT |
| R1.3 | 1 ATP:CYT + 1 F6P:CYT ⇒ 1 ADP:CYT + 1 F16P:CYT + 1 H:CYT |
| R1.4 | 1 F16P:CYT ⇒ 2 GAP:CYT |
| R1.5 | 1 GAP:CYT + 1 NAD:CYT + 1 PI:CYT ⇒ 1 13PG:CYT + 1 H:CYT + 1 NADH:CYT |
| R1.6 | 1 13PG:CYT + 1 ADP:CYT ⇒ 1 3PG:CYT + 1 ATP:CYT |
| R1.7 | 1 3PG:CYT ⇒ 1 2PG:CYT |
| R1.8 | 1 2PG:CYT ⇒ 1 H2O:CYT + 1 PEP:CYT |
| R1.9 | 1 ADP:CYT + 1 H:CYT + 1 PEP:CYT ⇒ 1 ATP:CYT + 1 PYR:CYT |
| R10.1 | 1 ADP:CYT + 1 ATP:MIT ⇒ 1 ADP:MIT + 1 ATP:CYT |
| R10.10 | 1 H:CYT + 1 ILE:MIT ⇒ 1 H:MIT + 1 ILE:CYT |
| R10.11 | 1 H:CYT + 1 PYR:CYT ⇒ 1 H:MIT + 1 PYR:MIT |
| R10.12 | 1 CO2:MIT ⇒ 1 CO2:CYT |
| R10.13 | 1 FUM:CYT + 1 H:CYT ⇒ 1 FUM:MIT + 1 H:MIT |
| R10.14 | 1 CITR:MIT + 1 MAL:CYT ⇒ 1 CITR:CYT + 1 MAL:MIT |
| R10.15 | 1 H:CYT + 1 THR:CYT ⇒ 1 H:MIT + 1 THR:MIT |
| R10.16 | 1 GLU:CYT + 1 H:CYT ⇒ 1 GLU:MIT + 1 H:MIT |
| R10.17 | 1 H:CYT + 1 VAL:MIT ⇒ 1 H:MIT + 1 VAL:CYT |
| R10.18 | 1 GLN:CYT + 1 H:CYT ⇒ 1 GLN:MIT + 1 H:MIT |
| R10.19 | 1 BIM:MIT + 1 H:CYT ⇒ 1 BIM:CYT + 1 H:MIT |
| R10.2 | 1 ACCARN:CYT + 1 CARN:MIT ⇒ 1 ACCARN:MIT + 1 CARN:CYT |
| R10.20 | 1 ACCOA:CYT + 1 CARN:CYT ⇒ 1 ACCARN:CYT + 1 HCOA:CYT |
| R10.21 | 1 ACCARN:MIT + 1 HCOA:MIT ⇒ 1 ACCOA:MIT + 1 CARN:MIT |
| R10.24 | 1 AKB:CYT ⇒ 1 AKB:MIT |
| R10.3 | 1 H2O:MIT ⇒ 1 H2O:CYT |
| R10.4 | 1 MAL:MIT + 1 SUCC:CYT ⇒ 1 MAL:CYT + 1 SUCC:MIT |
| R10.5 | 1 H:CYT + 1 PI:CYT ⇒ 1 H:MIT + 1 PI:MIT |
| R10.6 | 1 CITR:CYT + 1 ICITR:MIT ⇒ 1 CITR:MIT + 1 ICITR:CYT |
| R10.7 | 1 CTL:MIT + 1 H:CYT ⇒ 1 CTL:CYT + 1 H:MIT |
| R10.8 | 1 MAL:MIT + 1 PI:CYT ⇒ 1 MAL:CYT + 1 PI:MIT |
| R10.9 | 1 NH4:MIT ⇒ 1 NH4:CYT |
| R11.1 | 1 AKG:CYT + 1 H:CYT + 1 NADPH:CYT + 1 NH4:CYT ⇒ 1 GLU:CYT + 1 H2O:CYT + 1 NADP:CYT |
| R11.10 | 1 SER:CYT + 1 THF:CYT ⇒ 1 GLY:CYT + 1 H2O:CYT + 1 METHF:CYT |
| R11.11 | 2 ATP:CYT + 3 H:CYT + 1 H2O:CYT + 1 SO4:CYT ⇒ 1 ADP:CYT + 1 PAPS:CYT + 2 PI:CYT |
| R11.12 | 1 H:CYT + 4 NADPH:CYT + 1 PAPS:CYT ⇒ 1 ADP:CYT + 3 H2O:CYT + 1 H2S:CYT + 4 NADP:CYT |
| R11.13 | 1 ACCOA:CYT + 1 HOMSER:CYT ⇒ 1 ACHOMSER:CYT + 1 HCOA:CYT |
| R11.14 | 1 ACHOMSER:CYT + 1 H2S:CYT ⇒ 1 AC:CYT + 1 H:CYT + 1 HOMCYS:CYT |
| R11.16 | 1 GLU:CYT + 1 OAA:CYT ⇒ 1 AKG:CYT + 1 ASP:CYT |
| R11.17 | 1 ASP:CYT + 2 ATP:CYT + 1 H2O:CYT + 1 NH4:CYT ⇒ 2 ADP:CYT + 1 ASN:CYT + 2 H:CYT + 2 PI:CYT |
| R11.18 | 1 ASP:CYT + 1 ATP:CYT + 2 H:CYT + 2 NADPH:CYT ⇒ 1 ADP:CYT + 1 HOMSER:CYT + 2 NADP:CYT + 1 PI:CYT |
| R11.19 | 1 ATP:CYT + 1 H2O:CYT + 1 HOMSER:CYT ⇒ 1 ADP:CYT + 1 H:CYT + 1 PI:CYT + 1 THR:CYT |
| R11.2 | 1 ATP:CYT + 1 GLU:CYT + 1 NH4:CYT ⇒ 1 ADP:CYT + 1 GLN:CYT + 1 H:CYT + 1 PI:CYT |
| R11.20 | 1 HOMCYS:CYT + 1 MYTHF:CYT ⇒ 1 MET:CYT + 1 THF:CYT |
| R11.21 | 1 GLU:MIT + 2 H:MIT + 1 NADPH:MIT + 1 PYR:MIT + 1 THR:MIT ⇒ 1 AKG:MIT + 1 CO2:MIT + 1 H2O:MIT + 1 ILE:MIT + 1 NADP:MIT + 1 NH4:MIT |
| R11.22 | 1 GLU:CYT + 1 PYR:CYT ⇒ 1 AKG:CYT + 1 ALA:CYT |
| R11.23 | 2 H:MIT + 1 NADPH:MIT + 2 PYR:MIT ⇒ 1 AKI:MIT + 1 CO2:MIT + 1 H2O:MIT + 1 NADP:MIT |
| R11.24 | 1 AKI:MIT + 1 GLU:MIT ⇒ 1 AKG:MIT + 1 VAL:MIT |
| R11.25 | 1 ACCOA:MIT + 1 AKI:MIT + 1 H2O:MIT ⇒ 1 BIM:MIT + 1 H:MIT + 1 HCOA:MIT |
| R11.26 | 1 BIM:CYT + 1 GLU:CYT + 1 NAD:CYT ⇒ 1 AKG:CYT + 1 CO2:CYT + 1 LEU:CYT + 1 NADH:CYT |
| R11.27 | 1 ATP:CYT + 1 E4P:CYT + 1 NADPH:CYT + 2 PEP:CYT ⇒ 1 ADP:CYT + 1 CHOR:CYT + 1 NADP:CYT + 4 PI:CYT |
| R11.28 | 1 CHOR:CYT + 1 GLU:CYT + 1 H:CYT ⇒ 1 AKG:CYT + 1 CO2:CYT + 1 H2O:CYT + 1 PHE:CYT |
| R11.29 | 1 CHOR:CYT + 1 GLU:CYT + 1 NAD:CYT ⇒ 1 AKG:CYT + 1 CO2:CYT + 1 NADH:CYT + 1 TYR:CYT |
| R11.3 | 1 ATP:CYT + 1 GLU:CYT + 2 H:CYT + 2 NADPH:CYT ⇒ 1 ADP:CYT + 1 H2O:CYT + 2 NADP:CYT + 1 PI:CYT + 1 PRO:CYT |
| R11.30 | 1 CHOR:CYT + 1 GLN:CYT + 1 PRPP:CYT + 1 SER:CYT ⇒ 1 CO2:CYT + 1 GAP:CYT + 1 GLU:CYT + 1 H:CYT + 1 H2O:CYT + 2 PI:CYT + 1 PYR:CYT + 1 TRP:CYT |
| R11.31 | 2 ATP:CYT + 1 RIBU5P:CYT ⇒ 2 ADP:CYT + 1 H:CYT + 1 PRPP:CYT |
| R11.32 | 3 ATP:CYT + 1 CO2:CYT + 1 GLN:CYT + 3 H2O:CYT + 2 NAD:CYT + 1 NADPH:CYT + 1 NH4:CYT + 1 PRPP:CYT ⇒ |
|  | 3 ADP:CYT + 1 AKG:CYT + 8 H:CYT + 1 HIS:CYT + 2 NADH:CYT + 1 NADP:CYT + 6 PI:CYT |
| R11.33 | 0.137 ALA:CYT + 0.0273 ARG:CYT + 0.0358 ASN:CYT + 0.061 ASP:CYT + 0.00526 CYS:CYT + 0.175 GLN:CYT + 0.267 GLU:CYT + 0.0336 GLY:CYT + 0.0294 HIS:CYT + 0.0137 ILE:CYT + 0.0168 LEU:CYT + 0.0231 LYS:CYT + 0.00526 MET:CYT + 0.00526 PHE:CYT + 0.0578 PRO:CYT + 0.0494 SER:CYT + 0.0326 THR:CYT + 0.0021 TRP:CYT + 0.00631 TYR:CYT + 0.0168 |
| R11.34 | VAL:CYT ⇒ 4.56 AAPOOL:CYT 0.134 ALA:CYT + 0.07 ARG:CYT + 0.0386 ASN:CYT + 0.0386 ASP:CYT + 0.02 CYS:CYT + 0.0509 GLN:CYT + 0.0509 GLU:CYT +0.132 GLY:CYT + 0.0119 HIS:CYT + 0.0307 ILE:CYT + 0.0559 LEU:CYT + 0.0348 LYS:CYT + 0.0103 MET:CYT+ 0.0266 PHE:CYT + 0.0537 PRO:CYT + 0.044 SER:CYT + 0.0467 THR:CYT + 0.01 TRP:CYT + 0.0203 TYR:CYT+ 0.12 VAL:CYT ⇒ 4.53 EXPEPT:CYT + 1 H2O:CYT |
| R11.35 | 1 HOMCYS:CYT + 1 SER:CYT ⇒ 1 CYON:CYT + 1 H2O:CYT |
| R11.36 | 1 CYON:CYT + 1 H2O:CYT ⇒ 1 AKB:CYT + 1 CYS:CYT + 1 NH4:CYT |
| R11.37 | 1 AKB:MIT + 1 ATP:MIT + 1 H2O:MIT + 1 HCOA:MIT + 1 NAD:MIT ⇒ 1 ADP:MIT + 2 H:MIT + 1 NADH:MIT + 1 PI:MIT + 1 SUCCCOA:MIT |
| R11.4 | 2 ATP:MIT + 1 CO2:MIT + 1 GLN:MIT + 2 H2O:MIT ⇒ 2 ADP:MIT + 1 CARBP:MIT + 1 GLU:MIT + 3 H:MIT + 1 PI:MIT |
| R11.5 | 1 ATP:MIT + 1 CARBP:MIT + 2 GLU:MIT + 1 NADPH:MIT ⇒ 1 ADP:MIT + 1 AKG:MIT + 1 CTL:MIT + 1 H:MIT + 1 NADP:MIT + 2 PI:MIT |
| R11.6 | 1 ASP:CYT + 2 ATP:CYT + 1 CTL:CYT + 1 H2O:CYT ⇒ 2 ADP:CYT + 1 ARG:CYT + 1 FUM:CYT + 1 H:CYT + 2 PI:CYT |
| R11.7 | 1 ACCOA:CYT + 1 GLU:CYT + 1 H2O:CYT + 1 NAD:CYT ⇒ 1 AAD:CYT + 1 CO2:CYT + 1 HCOA:CYT + 1 NADH:CYT |
| R11.8 | 1 AAD:CYT + 2 ATP:CYT + 1 GLU:CYT + 1 H2O:CYT + 1 NAD:CYT + 2 NADPH:CYT ⇒ 2 ADP:CYT + 1 AKG:CYT + 1 H:CYT + 1 LYS:CYT + 1 NADH:CYT + 2 NADP:CYT + 2 PI:CYT |
| R11.9 | 1 3PG:CYT + 1 GLU:CYT + 1 H2O:CYT + 1 NAD:CYT ⇒ 1 AKG:CYT + 1 H:CYT + 1 NADH:CYT + 1 PI:CYT + 1 SER:CYT |
| R12.1 | 0.113 ALA:CYT + 0.0572 ARG:CYT + 0.038 ASN:CYT + 0.038 ASP:CYT + 0.00459 CYS:CYT + 0.0503 GLN:CYT + 0.0503 GLU:CYT + 0.102 GLY:CYT + 0.025 HIS:CYT + 0.0486 ILE:CYT + 0.0784 LEU:CYT + 0.0485 LYS:CYT + 0.0133 MET:CYT + + 0.0478 PHE:CYT + 0.0554 PRO:CYT + 0.0561 SER:CYT + 0.0608 THR:CYT + 0.0099 TRP:CYT + 0.0242 TYR:CYT + 0.0785 VAL:CYT ⇒ 1 AAPROTSYN:CYT |
| R12.2 | 1 AAPROTSYN:CYT + 4 ATP:CYT + 3 H2O:CYT ⇒ 4 ADP:CYT + 4 H:CYT + 4 PI:CYT + 4.81 PROT:CYT |
| R13.1 | 1 ASP:CYT + 4 ATP:CYT + 1 CO2:CYT + 2 FTHF:CYT + 2 GLN:CYT + 1 GLY:CYT + 2 H2O:CYT + 1 PRPP:CYT ⇒ 4 ADP:CYT + 1 FUM:CYT + 2 GLU:CYT + 8 H:CYT + 1 IMP:CYT + 6 PI:CYT + 2 THF:CYT |
| R13.2 | 1 ASP:CYT + 1 ATP:CYT + 1 IMP:CYT ⇒ 1 ADP:CYT + 1 AMP:CYT + 1 FUM:CYT + 2 H:CYT + 1 PI:CYT |
| R13.3 | 2 ATP:CYT + 1 GLN:CYT + 3 H2O:CYT + 1 IMP:CYT + 1 NAD:CYT ⇒ 2 ADP:CYT + 1 GLU:CYT + 1 GMP:CYT |
|  | + 4 H:CYT + 1 NADH:CYT + 2 PI:CYT |
| R13.4 | 1 ASP:CYT + 2 ATP:CYT + 1 GLN:CYT + 2 H2O:CYT + 1 NAD:CYT + 1 PRPP:CYT ⇒ 2 ADP:CYT + 1 GLU:CYT + 4 H:CYT + 1 NADH:CYT + 4 PI:CYT + 1 UMP:CYT |
| R13.5 | 2 ATP:CYT + 1 UMP:CYT ⇒ 2 ADP:CYT + 1 UTP:CYT |
| R13.6 | 1 ATP:CYT + 1 GLN:CYT + 1 H2O:CYT + 1 UTP:CYT ⇒ 1 ADP:CYT + 1 CTP:CYT + 1 GLU:CYT + 2 H:CYT + 1 PI:CYT |
| R13.7 | 2 ADP:CYT + 1 CTP:CYT ⇒ 2 ATP:CYT + 1 CMP:CYT |
| R13.8 | 1 A:CYT + 2 ATP:CYT ⇒ 3 ADP:CYT + 1 H:CYT |
| R13.9 | 1 ATP:CYT + 1 UDP:CYT ⇒ 1 ADP:CYT + 1 UTP:CYT |
| R14.1 | 0.349 AMP:CYT + 3.23 ATP:CYT + 0.168 CMP:CYT + 0.26 GMP:CYT + 2.23 H2O:CYT + 0.222 UMP:CYT ⇒ 3.23 ADP:CYT + 3.23 H:CYT + 3.23 PI:CYT + 9.61 RNA:CYT |
| R15.1 | 1 ATP:CYT + 1 H2O:CYT ⇒ 1 ADP:CYT + 1 H:CYT + 1 PI:CYT |
|  |  |
| R16.1 | 1 G6P:CYT + 1 H2O:CYT ⇒ 1 INO:CYT + 1 PI:CYT |
| R16.10 | 1 PHETA:CYT + 3 SAM:CYT ⇒ 3 H:CYT + 1 PHCHOL:CYT + 3 SAH:CYT |
| R16.11 | 1 CDPDACGCL:CYT + 1 INO:CYT ⇒ 1 CMP:CYT + 1 H:CYT + 1 PHINO:CYT |
| R16.12 | 1 H2O:CYT + 1 PHOSPHT:CYT + 1 STEACOA:CYT ⇒ 1 HCOA:CYT + 1 PI:CYT + 1 TRIA:CYT |
| R16.13 | 3 ACCOA:CYT + 1 H:CYT + 1 H2O:CYT + 2 NADPH:CYT ⇒ 3 HCOA:CYT + 1 MEVA:CYT + 2 NADP:CYT |
| R16.14 | 18 ATP:CYT + 5 H2O:CYT + 6 MEVA:CYT + 2 NADPH:CYT + 1 O2:CYT ⇒ 18 ADP:CYT + 6 CO2:CYT + 10 H:CYT + 1 LANO:CYT + 2 NADP:CYT + 18 PI:CYT |
| R16.15 | 1 LANO:CYT + 1 NAD:CYT + 2 THF:CYT ⇒ 1 ERGO:CYT + 1 H:CYT + 2 MYTHF:CYT + 1 NADH:CYT |
| R16.16 | 1 ERGO:CYT + 1 OLCOA:CYT ⇒ 1 ESE:CYT + 1 HCOA:CYT |
| R16.2 | 1 GAP:CYT + 1 H:CYT + 1 NADH:CYT ⇒ 1 GCL3P:CYT + 1 NAD:CYT |
| R16.3 | 9 ACCOA:CYT + 8 ATP:CYT + 8 H:CYT + 16 NADPH:CYT ⇒ 8 ADP:CYT + 8 HCOA:CYT + 16 NADP:CYT + 8 PI:CYT + 1 STEACOA:CYT |
| R16.4 | 1 H:CYT + 1 NADH:CYT + 1 O2:CYT + 1 STEACOA:CYT ⇒ 2 H2O:CYT + 1 NAD:CYT + 1 OLCOA:CYT |
| R16.5 | 1 H:CYT + 1 NADH:CYT + 1 O2:CYT + 1 OLCOA:CYT ⇒ 2 H2O:CYT + 1 LINCOA:CYT + 1 NAD:CYT |
| R16.6 | 1 GCL3P:CYT + 1 LINCOA:CYT + 1 OLCOA:CYT ⇒ 2 HCOA:CYT + 1 PHOSPHT:CYT |
| R16.7 | 1 CTP:CYT + 1 H2O:CYT + 1 PHOSPHT:CYT ⇒ 1 CDPDACGCL:CYT + 2 PI:CYT |
| R16.8 | 1 CDPDACGCL:CYT + 1 SER:CYT ⇒ 1 CMP:CYT + 1 H:CYT + 1 PHSER:CYT |
| R16.9 | 1 H:CYT + 1 PHSER:CYT ⇒ 1 CO2:CYT + 1 PHETA:CYT |
| R17.1 | 0.167 ATP:CYT + 0.167 G6P:CYT + 0.167 H2O:CYT ⇒ 0.167 ADP:CYT + 0.167 H:CYT + 0.333 PI:CYT + 1 PSACCH:CYT |
| R17.2 | 1 ACCOA:CYT + 1 F6P:CYT + 1 GLN:CYT ⇒ 1 CHIT:CYT + 1 GLU:CYT + 1 H:CYT + 1 HCOA:CYT + 1 PI:CYT |
| R17.3 | 1 F6P:CYT + 1 H:CYT + 1 NADH:CYT ⇒ 1 M1P:CYT + 1 NAD:CYT |
| R17.4 | 1 H2O:CYT + 1 M1P:CYT ⇒ 1 MAN:CYT + 1 PI:CYT |
| R17.5 | 1 G6P:CYT + 1 H2O:CYT + 1 UTP:CYT ⇒ 2 PI:CYT + 1 UDPGLC:CYT |
| R17.6 | 1 G6P:CYT + 1 UDPGLC:CYT ⇒ 1 H:CYT + 1 T6P:CYT + 1 UDP:CYT |
| R17.7 | 1 H2O:CYT + 1 T6P:CYT ⇒ 1 PI:CYT + 1 TRE:CYT |
| R17.8 | 1 E4P:CYT + 1 H:CYT + 1 H2O:CYT + 1 NADH:CYT ⇒ 1 ERY:CYT + 1 NAD:CYT + 1 PI:CYT |
| R18.18 | 0.0364 AAPOOL:CYT + 0.00805 CHIT:CYT + 0.000927 ERY:CYT + 0.00047 ESE:CYT + 0.076 H2O:CYT + 0.00354 MAN:CYT + 0.000939 PHCHOL:CYT + 0.000564 PHETA:CYT + 0.000376 PHINO:CYT + 0.449 PROT:CYT + 0.249 PSACCH:CYT + 0.0568 RNA:CYT + 0.000237 TRE:CYT + 0.000235 TRIA:CYT ⇒ 1 BIOM_CC_ETOH:EXT |
| R18.3 | 0.0344 AAPOOL:CYT + 0.0083 CHIT:CYT + 0.00124 ERY:CYT + 0.000503 ESE:CYT + 0.0801 H2O:CYT + 0.00247 MAN:CYT + 0.00101 PHCHOL:CYT + 0.000604 PHETA:CYT + 0.000403 PHINO:CYT + 0.425 PROT:CYT + 0.266 PSACCH:CYT + 0.0514 RNA:CYT + 0.00107 TRE:CYT + 0.000252 TRIA:CYT ⇒ 1 BIOM_CC6:EXT |
| R19.1 | 1 AAD:CYT + 6 ATP:CYT + 1 CYS:CYT + 4 H2O:CYT + 1 VAL:CYT ⇒ 1 ACV:CYT + 6 ADP:CYT + 6 H:CYT + 6 PI:CYT |
| R19.2 | 1 ACV:CYT + 1 O2:CYT ⇒ 2 H2O:CYT + 1 IPN:CYT |
| R19.3 | 2 ATP:PER + 1 H2O:PER + 1 HCOA:PER + 1 PAA:PER ⇒ 2 ADP:PER + 2 H:PER + 1 PAACOA:PER + 2 PI:PER |
| R19.4 | 1 H2O:PER + 1 IPN:PER + 1 PAACOA:PER ⇒ 1 AAD:PER + 1 HCOA:PER + 1 PENG:PER |
| R2.1 | 1 G6P:CYT + 1 H2O:CYT + 1 NADP:CYT ⇒ 1 6PGLUCT:CYT + 2 H:CYT + 1 NADPH:CYT |
| R2.2 | 1 6PGLUCT:CYT + 1 NADP:CYT ⇒ 1 CO2:CYT + 1 NADPH:CYT + 1 RIBU5P:CYT |
| R2.3 | 1 RIBU5P:CYT ⇒ 1 RIB5P:CYT |
| R2.4 | 1 RIBU5P:CYT ⇒ 1 XYLU5P:CYT |
| R2.5 | 1 RIB5P:CYT + 1 XYLU5P:CYT ⇒ 1 GAP:CYT + 1 SED7P:CYT |
| R2.6 | 1 GAP:CYT + 1 SED7P:CYT ⇒ 1 E4P:CYT + 1 F6P:CYT |
| R2.7 | 1 E4P:CYT + 1 XYLU5P:CYT ⇒ 1 F6P:CYT + 1 GAP:CYT |
| R21.1 | 1 PAA:CYT ⇒ 1 PAA:PER |
| R21.10 | 1 ACCARN:PER + 1 HCOA:PER ⇒ 1 ACCOA:PER + 1 CARN:PER |
| R21.11 | 1 H:PER + 1 MAL:PER ⇒ 1 H:CYT + 1 MAL:CYT |
| R21.12 | 1 ATP:CYT + 1 H2O:CYT ⇒ 1 ADP:CYT + 1 H:PER + 1 PI:CYT |
| R21.13 | 1 ADP:PER + 1 ATP:CYT ⇒ 1 ADP:CYT + 1 ATP:PER |
| R21.14 | 1 H2O:PER ⇒ 1 H2O:CYT |
| R21.15 | 1 H:PER + 1 PI:PER ⇒ 1 H:CYT + 1 PI:CYT |
| R21.2 | 1 IPN:CYT ⇒ 1 IPN:PER |
| R21.6 | 1 AAD:PER ⇒ 1 AAD:CYT |
| R21.7 | 1 PENG:PER ⇒ 1 PENG:CYT |
| R21.8 | 1 ICITR:CYT + 1 SUCC:PER ⇒ 1 ICITR:PER + 1 SUCC:CYT |
| R21.9 | 1 ACCARN:CYT + 1 CARN:PER ⇒ 1 ACCARN:PER + 1 CARN:CYT |
| R3.1 | 1 ATP:CYT + 1 OAA:CYT ⇒ 1 ADP:CYT + 1 CO2:CYT + 1 PEP:CYT |
| R4.10 | 1 FUM:MIT + 1 H2O:MIT ⇒ 1 MAL:MIT |
| R4.11 | 1 MAL:MIT + 1 NAD:MIT ⇒ 1 H:MIT + 1 NADH:MIT + 1 OAA:MIT |
|  |  |
| R4.2 | 1 ACCOA:MIT + 1 H2O:MIT + 1 OAA:MIT ⇒ 1 CITR:MIT + 1 H:MIT + 1 HCOA:MIT |
| R4.3 | 1 CITR:MIT ⇒ 1 ICITR:MIT |
| R4.4 | 1 ICITR:MIT + 1 NAD:MIT ⇒ 1 AKG:MIT + 1 CO2:MIT + 1 NADH:MIT |
| R4.5 | 1 ICITR:MIT + 1 NADP:MIT ⇒ 1 AKG:MIT + 1 CO2:MIT + 1 NADPH:MIT |
| R4.6 | 1 ICITR:CYT + 1 NADP:CYT ⇒ 1 AKG:CYT + 1 CO2:CYT + 1 NADPH:CYT |
| R4.7 | 1 AKG:MIT + 1 HCOA:MIT + 1 NAD:MIT ⇒ 1 CO2:MIT + 1 NADH:MIT + 1 SUCCCOA:MIT |
| R4.8 | 1 ADP:MIT + 1 PI:MIT + 1 SUCCCOA:MIT ⇒ 1 ATP:MIT + 1 HCOA:MIT + 1 SUCC:MIT |
| R4.9 | 1 FAD:MIT + 1 SUCC:MIT ⇒ 1 FADH2:MIT + 1 FUM:MIT |
| R5.1 | 1 ATP:CYT + 1 CO2:CYT + 1 H2O:CYT + 1 PYR:CYT ⇒ 1 ADP:CYT + 2 H:CYT + 1 OAA:CYT + 1 PI:CYT |
| R5.3 | 1 H:CYT + 1 NADH:CYT + 1 OAA:CYT ⇒ 1 MAL:CYT + 1 NAD:CYT |
| R5.4 | 1 ICITR:PER ⇒ 1 GLYOX:PER + 1 SUCC:PER |
| R5.5 | 1 ACCOA:PER + 1 GLYOX:PER + 1 H2O:CYT ⇒ 1 H:CYT + 1 HCOA:PER + 1 MAL:PER |
| R6.1B | 8.39 H:MIT + 1 NADH:MIT + 0.5 O2:CYT ⇒ 7.39 H:CYT + 1 H2O:CYT + 1 NAD:MIT |
| R6.2B | 5.44 H:MIT + 1 NADH:CYT + 0.5 O2:CYT ⇒ 4.44 H:CYT + 1 H2O:CYT + 1 NAD:CYT |
| R6.3B | 1 FADH2:MIT + 4.44 H:MIT + 0.5 O2:CYT ⇒ 1 FAD:MIT + 4.44 H:CYT + 1 H2O:CYT |
| R6.4 | 1 ADP:MIT + 4 H:CYT + 1 PI:MIT ⇒ 1 ATP:MIT + 3 H:MIT + 1 H2O:MIT |
| R7.1 | 1 ETOH:CYT + 1 NAD:CYT ⇒ 1 AALD:CYT + 1 H:CYT + 1 NADH:CYT |
| R7.2 | 1 AC:CYT + 2 ATP:CYT + 1 H2O:CYT + 1 HCOA:CYT ⇒ 1 ACCOA:CYT + 2 ADP:CYT + 1 H:CYT + 2 PI:CYT |
| R7.3 | 1 AALD:CYT + 1 H2O:CYT + 1 NAD:CYT ⇒ 1 AC:CYT + 2 H:CYT + 1 NADH:CYT |
| R8.1 | 1 GLY:CYT + 1 NAD:CYT + 1 THF:CYT ⇒ 1 CO2:CYT + 1 METHF:CYT + 1 NADH:CYT + 1 NH4:CYT |
| R8.2 | 1 H:CYT + 1 METHF:CYT + 1 NADH:CYT ⇒ 1 MYTHF:CYT + 1 NAD:CYT |
| R8.3 | 1 ATP:CYT + 2 H2O:CYT + 1 METHF:CYT + 1 NAD:CYT ⇒ 1 ADP:CYT + 1 FTHF:CYT + 2 H:CYT + 1 NADH:CYT + 1 PI:CYT |
| R8.4 | 1 ATP:CYT + 2 H2O:CYT + 1 MET:CYT ⇒ 1 H:CYT + 3 PI:CYT + 1 SAM:CYT |
| R8.5 | 1 H2O:CYT + 1 SAH:CYT ⇒ 1 A:CYT + 1 HOMCYS:CYT |
| R9.1 | 1 ATP:CYT + 1 H2O:CYT ⇒ 1 ADP:CYT + 1 H:EXT + 1 PI:CYT |
| R9.11 | 1 PAA:EXT ⇒ 1 PAA:CYT |
| R9.13 | 1 EXPEPT:CYT ⇒ 1 EXPEPT:EXT |
| R9.14 | 1 PSACCH:CYT ⇒ 1 PSACCH:EXT |
| R9.15 | 1 H2O:CYT ⇒ 1 H2O:EXT |
| R9.18 | 1 ETOH:EXT ⇒ 1 ETOH:CYT |
| R9.2 | 2 H:EXT + 1 PI:EXT ⇒ 2 H:CYT + 1 PI:CYT |
| R9.20 | 1 H:EXT + 1 NH4:EXT ⇒ 1 H:CYT + 1 NH4:CYT |
| R9.3 | 1 GLC:EXT + 1 H:EXT ⇒ 1 GLC:CYT + 1 H:CYT |
| R9.4 | 2 H:EXT + 1 SO4:EXT ⇒ 2 H:CYT + 1 SO4:CYT |
| R9.6 | 1 O2:EXT ⇒ 1 O2:CYT |
| R9.7 | 1 CO2:CYT ⇒ 1 CO2:EXT |
| R9.8 | 1 PENG:CYT ⇒ 1 PENG:EXT |
