# Supplementary material for: Substrate cycles in Penicillium chrysogenum quantified by isotopic non-stationary flux analysis
Source: Microb Cell Fact. 2012 Oct 25;11:140. doi: 10.1186/1475-2859-11-140 (PMC3538697; doi:10.1186/1475-2859-11-140)
Supplement: Additional file 3 — Table S4. Metabolic reaction network with atom transitions. [file 1475-2859-11-140-S3.docx]

**Table S4**: Metabolic reaction network with atom transitions.

| Name | Reaction |  |  |  |
| --- | --- | --- | --- | --- |
| rLUGlc: | LUGlc | > | GLC_ext |  |
|  | #ABCDEF | > | #ABCDEF |  |
| rL1Glc: | L1Glc | > | GLC_ext |  |
|  | #ABCDEF | > | #ABCDEF |  |
| rLNGlc: | LNGlc | > | GLC_ext |  |
|  | #ABCDEF | > | #ABCDEF |  |
| rLUEth: | LUEtOH | > | EtOH_ext |  |
|  | #AB | > | #AB |  |
| rLNEth: | LNEtOH | > | EtOH_ext |  |
|  | #AB | > | #AB |  |
| r1_1: | GLC_c | > | G6P_c |  |
|  | #ABCDEF | > | #ABCDEF |  |
| r1_2: | G6P_c | <> | F6P_c |  |
|  | #ABCDEF | > | #ABCDEF |  |
| r1_3: | F6P_c | > | FBP_c |  |
|  | #ABCDEF | > | #ABCDEF |  |
| r1_4: | FBP_c | <> | DHAP_c + GAP_c |  |
|  | #ABCDEF | > | #CBA + #DEF |  |
| r1_4b: | DHAP_c | <> | GAP_c |  |
|  | #ABC | > | #ABC |  |
| r1_5: | GAP_c | <> | BPG_c |  |
|  | #ABC | > | #ABC |  |
| r1_6: | BPG_c | <> | PG3_c |  |
|  | #ABC | > | #ABC |  |
| r1_7: | PG3_c | <> | PG2_c |  |
|  | #ABC | > | #ABC |  |
| r1_8: | PG2_c | <> | PEP_c |  |
|  | #ABC | > | #ABC |  |
| r1_9: | PEP_c | > | PYR_c |  |
|  | #ABC | > | #ABC |  |
| r2_1: | G6P_c | > | PG6_c |  |
|  | #ABCDEF | > | #ABCDEF |  |
| r2_2: | PG6_c | > | CO2_c + Rib5P_c |  |
|  | #ABCDEF | > | #A + #BCDEF |  |
| r2_3: | Rib5P_c | <> | R5P_c |  |
|  | #ABCDE | > | #ABCDE |  |
| r2_4: | Rib5P_c | <> | Xylu5P_c |  |
|  | #ABCDE | > | #ABCDE |  |
| r2_5: | R5P_c + Xylu5P_c | <> | Sed7P_c + GAP_c |  |
|  | #ABCDE + #abcde | > | #ABabcde + #CDE |  |
| r2_6: | GAP_c + Sed7P_c | <> | E4P_c + F6P_c |  |
|  | #ABC + #abcdefg | > | #defg + #abcABC |  |
| r2_7: | E4P_c + Xylu5P_c | <> | F6P_c + GAP_c |  |
|  | #abcd + #ABCDE | > | #ABabcd + #CDE |  |
| r3_1: | OAA_c | > | PEP_c + CO2_c |  |
|  | #ABCD | > | #ABC + #D |  |
| r3_2: | FBP_c | > | F6P_c |  |
|  | #ABCDEF | > | #ABCDEF |  |
| r4_1: | PYR_m | > | AcCoA_m + CO2_m |  |
|  | #ABC | > | #BC + #A |  |
| r4_2: | AcCoA_m + OAA_m | > | CITR_m |  |
|  | #AB + #abcd | > | #dcbaBA |  |
| r4_3: | CITR_m | <> | ICITR_m |  |
|  | #ABCDEF | > | #ABCDEF |  |
| r4_4: | ICITR_m | > | AKG_m + CO2_m |  |
|  | #ABCDEF | > | #ABCEF + #D |  |
| r4_6: | ICITR_c | > | AKG_c + CO2_c |  |
|  | #ABCDEF | > | #ABCEF + #D |  |
| r4_7x: | AKG_m | > | SUCC_m + CO2_m |  |
|  | #ABCDE | > | #BCDE + #A |  |
| r4_7y: | AKG_m | > | SUCC_m + CO2_m |  |
|  | #ABCDE | > | #EDCB + #A |  |
| r4_9x: | SUCC_m | <> | FUM_m |  |
|  | #ABCD | > | #ABCD |  |
| r4_9y: | SUCC_m | <> | FUM_m |  |
|  | #ABCD | > | #DCBA |  |
| r4_10: | FUM_m | <> | MAL_m |  |
|  | #ABCD | > | #ABCD |  |
| r4_11: | MAL_m | <> | OAA_m |  |
|  | #ABCD | > | #ABCD |  |
| r5_1: | PYR_c + CO2_c | > | OAA_c |  |
|  | #ABC + #a | > | #ABCa |  |
| r5_2: | CITR_c | > | AcCoA_c + OAA_c |  |
|  | #ABCDEF | > | #FE + #DCBA |  |
| r5_3: | OAA_c | <> | MAL_c |  |
|  | #ABCD | > | #ABCD |  |
| r5_4: | ICITR_p | > | GLYOX_p + SUCC_p |  |
|  | #ABCDEF | > | #AB + #DCEF |  |
| r5_5: | AcCoA_p + GLYOX_p | > | MAL_p |  |
|  | #AB + #ab | > | #abBA |  |
| r7_1: | EtOH_c | > | Ac_c |  |
|  | #AB | > | #AB |  |
| r7_2: | Ac_c | > | AcCoA_c |  |
|  | #AB | > | #AB |  |
| r8_1: | FTHF_c + CO2_c | > | GLY_c |  |
|  | #B + #A | > | #AB |  |
| r8_4: | MET_c | > | HOMCYS_c + MYTHF_c |  |
|  | #ABCDE | > | #ABCD + #E |  |
| r9_3: | GLC_ext | > | GLC_c |  |
|  | #ABCDEF | > | #ABCDEF |  |
| r9_7: | CO2_c | > | CO2_ext |  |
|  | #A | > | #A |  |
| r9_14: | PSacch_c | > | PSacch_ext |  |
|  | #ABCDEF | > | #ABCDEF |  |
| r9_18: | EtOH_ext | > | EtOH_c |  |
|  | #AB | > | #AB |  |
| r10_4: | MAL_m + SUCC_c | <> | MAL_c + SUCC_m |  |
|  | #ABCD + #abcd | > | #ABCD + #abcd |  |
| r10_6: | CITR_c + ICITR_m | <> | CITR_m + ICITR_c |  |
|  | #ABCDEF + #abcdef | > | #ABCDEF + #abcdef |  |
| r10_8: | MAL_m | > | MAL_c |  |
|  | #ABCD | > | #ABCD |  |
| r10_10: | ILE_m | > | ILE_c |  |
|  | #ABCDEF | > | #ABCDEF |  |
| r10_11: | PYR_c | > | PYR_m |  |
|  | #ABC | > | #ABC |  |
| r10_12: | CO2_m | > | CO2_c |  |
|  | #A | > | #A |  |
| r10_13: | FUM_c | > | FUM_m |  |
|  | #ABCD | > | #ABCD |  |
| r10_14: | CITR_m + MAL_c | <> | CITR_c + MAL_m |  |
|  | #ABCDEF + #abcd | > | #ABCDEF + #abcd |  |
| r10_15: | THR_c | > | THR_m |  |
|  | #ABCD | > | #ABCD |  |
| r10_16: | GLU_c | > | GLU_m |  |
|  | #ABCDE | > | #ABCDE |  |
| r10_17: | VAL_m | > | VAL_c |  |
|  | #ABCDE | > | #ABCDE |  |
| r10_18: | GLN_c | > | GLN_m |  |
|  | #ABCDE | > | #ABCDE |  |
| r10_19: | bIM_m | > | bIM_c |  |
|  | #ABCDEF | > | #ABCDEF |  |
| r10_21: | AcCoA_c | > | AcCoA_m |  |
|  | #AB | > | #AB |  |
| r11_1: | AKG_c | <> | GLU_c |  |
|  | #ABCDE | > | #ABCDE |  |
| r11_2: | GLU_c | <> | GLN_c |  |
|  | #ABCDE | > | #ABCDE |  |
| r11_3: | GLU_c | > | PRO_c |  |
|  | #ABCDE | > | #ABCDE |  |
| r11_4: | GLN_m + CO2_m | > | AKG_m + CarbP_m |  |
|  | #ABCDE + #1 | > | #ABCDE + #1 |  |
| r11_5: | CarbP_m + glu_m | > | CTL_c |  |
|  | #1 + #ABCDE | > | #ABCDE1 |  |
| r11_6: | CTL_c + ASP_c | > | ARG_c + FUM_c |  |
|  | #ABCDEF + #abcd | > | #ABCDEF + #abcd |  |
| r11_7: | AcCoA_c + GLU_c | > | AAD_c + CO2_c |  |
|  | #A1 + #abcde | > | #A1bcde + #a |  |
| r11_8: | AAD_c + GLU_c | > | LYS_c + AKG_c |  |
|  | #ABCDEF + #abcde | > | #ABCDEF + #abcde |  |
| r11_9: | PG3_c + GLU_c | > | SER_c + AKG_c |  |
|  | #ABC + #abcde | > | #ABC + #abcde |  |
| r11_10: | SER_c | > | GLY_c + FTHF_c |  |
|  | #ABC | > | #AB + #C |  |
| r11_13: | AcCoA_c + HOMSER_c | > | HOMCYS_c + Ac_c |  |
|  | #ab + #ABCD | > | #ABCD + #ab |  |
| r11_15: | AcCoA_c + SER_c | > | Ac_c + CYS_c |  |
|  | #AB + #abc | > | #AB + #abc |  |
| r11_16: | OAA_c + GLU_c | <> | ASP_c + AKG_c |  |
|  | #ABCD + #abcde | > | #ABCD + #abcde |  |
| r11_17: | ASP_c | <> | ASN_c |  |
|  | #ABCD | > | #ABCD |  |
| r11_18: | ASP_c | > | HOMSER_c |  |
|  | #ABCD | > | #ABCD |  |
| r11_19: | HOMSER_c | > | THR_c |  |
|  | #ABCD | > | #ABCD |  |
| r11_20: | HOMCYS_c + FTHF_c | > | MET_c |  |
|  | #ABCD + #a | > | #ABCDa |  |
| r11_21a: | THR_m + PYR_m | > | ILEA_m + CO2_m |  |
|  | #ABCD + #abc | > | #ABbCDc + #a |  |
| r11_21b: | ILEA_m + GLU_m | > | ILE_m + AKG_m |  |
|  | #ABCDEF + #abcde | > | #ABCDEF + #abcde |  |
| r11_22: | PYR_c + GLU_c | <> | ALA_c + AKG_c |  |
|  | #ABC + #abcde | > | #ABC + #abcde |  |
| r11_23: | PYR_m + PYR_m | > | AKI_m + CO2_m |  |
|  | #ABC + #abc | > | #ABbcC + #a |  |
| r11_24: | AKI_m + GLU_m | > | AKG_m + VAL_m |  |
|  | #ABCDE + #abcde | > | #abcde + #ABCDE |  |
| r11_25: | AKI_m + AcCoA_c | > | bIM_m + CO2_c |  |
|  | #ABCDE + #ab | > | #abBCDE + #A |  |
| r11_26: | bIM_c + glu_c | > | LEU_c + AKG_c |  |
|  | #ABCDEF + #abcde | > | #ABCDEF + #abcde |  |
| r11_32a: | Rib5P_c + CO2_c | > | HISA_c |  |
|  | #ABCDE + #a | > | #EDCBAa |  |
| r11_32b: | HISA_c + GLN_c | > | HIS_c + AKG_c |  |
|  | #ABCDEF + #abcde | > | #ABCDEF + #abcde |  |
| r11_35: | HOMCYS_c + SER_c | > | CYS_c + SUCC_m |  |
|  | #ABCD + #abc | > | #abc + #ABCD |  |
| r16_9: | SER_c | > | PhetaA_c + CO2_c |  |
|  | #ABC | > | #BC + #A |  |
| r16_13: | AcCoA_c | > | ESEA_c + CO2_c |  |
|  | #AB | > | #B + #A |  |
| r17_1: | G6P_c | <> | PSacch_c |  |
|  | #ABCDEF | > | #ABCDEF |  |
| r21_8: | ICITR_c + SUCC_p | > | ICITR_p + SUCC_c |  |
|  | #ABCDEF + #abcd | > | #ABCDEF + #abcd |  |
| r21_10: | AcCoA_c | > | AcCoA_p |  |
|  | #AB | > | #AB |  |
| r21_11: | MAL_p | > | MAL_c |  |
|  | #abcd | > | #abcd |  |
| bm_ala: | ALA_c | > | ALAbio |  |
|  | #ABC | > | #ABC |  |
| bm_arg: | ARG_c | > | ARGbio |  |
|  | #ABCDEF | > | #ABCDEF |  |
| bm_asn: | ASN_c | > | ASNbio |  |
|  | #ABCD | > | #ABCD |  |
| bm_asp: | ASP_c | > | ASPbio |  |
|  | #ABCD | > | #ABCD |  |
| bm_cys: | CYS_c | > | CYSbio |  |
|  | #ABC | > | #ABC |  |
| bm_gln: | GLN_c | > | GLNbio |  |
|  | #ABCDE | > | #ABCDE |  |
| bm_glu: | GLU_c | > | GLUbio |  |
|  | #ABCDE | > | #ABCDE |  |
| bm_akg: | GLU_c | > | AKG_c |  |
|  | #ABCDE | > | #ABCDE |  |
| bm_glnglu: | GLN_c | > | GLU_c |  |
|  | #ABCDE | > | #ABCDE |  |
| bm_gly: | GLY_c | > | GLYbio |  |
|  | #AB | > | #AB |  |
| bm_his: | HIS_c | > | HISbio |  |
|  | #ABCDEF | > | #ABCDEF |  |
| bm_ile: | ILE_c | > | ILEbio |  |
|  | #ABCDEF | > | #ABCDEF |  |
| bm_leu: | LEU_c | > | LEUbio |  |
|  | #ABCDEF | > | #ABCDEF |  |
| bm_lys: | LYS_c | > | LYSbio |  |
|  | #ABCDEF | > | #ABCDEF |  |
| bm_met: | MET_c | > | METbio |  |
|  | #ABCDE | > | #ABCDE |  |
| bm_pro: | PRO_c | > | PRObio |  |
|  | #ABCDE | > | #ABCDE |  |
| bm_ser: | SER_c | > | SERbio |  |
|  | #ABC | > | #ABC |  |
| bm_thr: | THR_c | > | THRbio |  |
|  | #ABCD | > | #ABCD |  |
| bm_val: | VAL_c | > | VALbio |  |
|  | #ABCDE | > | #ABCDE |  |
| bm_g6p: | G6P_c | > | G6Pbio |  |
|  | #ABCDEF | > | #ABCDEF |  |
| bm_f6p: | F6P_c | > | F6Pbio |  |
|  | #ABCDEF | > | #ABCDEF |  |
| bm_prpp: | Rib5P_c | > | PRPPbio |  |
|  | #ABCDE | > | #ABCDE |  |
| bm_e4p0: | E4P_c | > | E4Pbio |  |
|  | #ABCD | > | #ABCD |  |
| bm_e4p: | E4P_c | > | E4Pbio2 + CO2_c |  |
|  | #ABCD | > | #ABC + #D |  |
| bm_gap: | GAP_c | > | GAPbio |  |
|  | #ABC | > | #ABC |  |
| bm_accoa: | AcCoA_c | > | AcCoAbio |  |
|  | #AB | > | #AB |  |
| bm_aspfum: | ASP_c | > | FUM_c |  |
|  | #ABCD | > | #ABCD |  |
| bm_fthf: | FTHF_c | > | FTHFbio |  |
|  | #A | > | #A |  |
| bm_co2: | CO2_c | > | CO2bio2 |  |
|  | #A | > | #A |  |
| bm_psacch: | PSacch_c | > | PSacchbio |  |
|  | #ABCDEF | > | #ABCDEF |  |
| bm_mythf: | MYTHF_c | > | MYTHFbio |  |
|  | #A | > | #A |  |
| bm_phetaA: | PhetaA_c | > | PhetaAbio |  |
|  | #AB | > | #AB |  |
| bm_eseA: | ESEA_c | > | ESEAbio |  |
|  | #A | > | #A |  |
| bm_pep: | PEP_c | > | PEPbio |  |
|  | #ABC | > | #ABC |  |
| rz_1: | G6P_c | > | TRE6P_c |  |
|  | #ABCDEF | > | #ABCDEF |  |
| rz_2a: | TRE6P_c | > | TRE_c |  |
|  | #ABCDEF | > | #ABCDEF |  |
| rz_2b: | TRE_c | > | TRE_ext |  |
|  | #ABCDEF | > | #ABCDEF |  |
| rz_3: | TRE_c | > | GLC_c |  |
|  | #ABCDEF | > | #ABCDEF |  |
| rz_3b: | TRE_ext | > | GLC_ext |  |
|  | #ABCDEF | > | #ABCDEF |  |
| bm_pep2: | PEPbio | > | PEPbio2 |  |
|  | #ABC | > | #ABC |  |
| bm_pyr: | PEPbio | > | PYR_c |  |
|  | #ABC | > | #ABC |  |
| bm_e4p1: | E4Pbio2 | > | E4Pbio4 |  |
|  | #ABC | > | #ABC |  |
| rz_4a: | F6P_c | <> | Manol_c |  |
|  | #ABCDEF | > | #ABCDEF |  |
| rz_4a2: | F6P_c | <> | Manol_c |  |
|  | #ABCDEF | > | #FEDCBA |  |
| rz_4b: | Manol_c | <> | Manol_ext |  |
|  | #ABCDEF | > | #ABCDEF |  |
| rz_4c: | Manol_c | > | manolbio |  |
|  | #ABCDEF | > | #ABCDEF |  |
| rz_5a: | E4P_c | <> | Erytol_c |  |
|  | #ABCD | > | #ABCD |  |
| rz_5b: | Erytol_c | <> | Erytol_ext |  |
|  | #ABCD | > | #ABCD |  |
| rz_6a: | R5P_c | <> | Rbtol_c |  |
|  | #ABCDE | > | #ABCDE |  |
| rz_6b: | Rbtol_c | <> | Rbtol_ext |  |
|  | #ABCDE | > | #ABCDE |  |
| rz_6c: | R5P_c | <> | RNA |  |
|  | #ABCDE | > | #ABCDE |  |
| rz_7: | FUM_c | <> | MAL_c |  |
|  | #ABCD | > | #ABCD |  |
| rz_7b: | SUCC_c | <> | FUM_c |  |
|  | #ABCD | > | #ABCD |  |
| rz_7c: | SUCC_c + FUM_m | <> | SUCC_m + FUM_c |  |
|  | #ABCD + #abcd | > | #ABCD + #abcd |  |
| rz_8a: | ul_PYR | > | PYR_m |  |
|  | #ABC | > | #ABC |  |
| rz_8b: | PYR_m | > | PYR_ext |  |
|  | #ABC | > | #ABC |  |
| rz_8c1: | GLU_c | > | SUCC_c + CO2_c |  |
|  | #ABCDE | > | #BCDE + #A |  |
| rz_8d1: | ul_AcCoA | > | AcCoA_c |  |
|  | #AB | > | #AB |  |
| rz_8d2: | AcCoA_c | > | AcCoA_ext |  |
|  | #AB | > | #AB |  |
| rz_9: | G6P_c | > | GLC_c |  |
|  | #ABCDEF | > | #ABCDEF |  |
| rz_9b: | GLC_c | > | GLC_ext |  |
|  | #ABCDEF | > | #ABCDEF |  |
| rz_9c: | PSacch_c | > | GLC_c |  |
|  | #ABCDEF | > | #ABCDEF |  |
| rz_9c2: | PSacch_c | > | G6P_c |  |
|  | #ABCDEF | > | #ABCDEF |  |
| rz_9d: | TRE_c | > | TREbio |  |
|  | #ABCDEF | > | #ABCDEF |  |
