# Supplementary material for: Substrate cycles in Penicillium chrysogenum quantified by isotopic non-stationary flux analysis
Source: Microb Cell Fact. 2012 Oct 25;11:140. doi: 10.1186/1475-2859-11-140 (PMC3538697; doi:10.1186/1475-2859-11-140)
Supplement: Additional file 4 — Table S2. Metabolic network used for the metabolic flux analysis (unlabeled). [file 1475-2859-11-140-S4.docx]

Table A6: Measured mass isotopomer ratios.

|  |  | Time (h) | | | | | |
| --- | --- | --- | --- | --- | --- | --- | --- |
| Metabolite | m/z | 0.033 | 0.067 | 0.133 | 0.267 | 0.533 | 1.067 |
| **AKG** | +0 | 0.573 | 0.393 | 0.514 | 0.404 | 0.353 | 0.291 |
|  | +1 | 0.144 | 0.146 | 0.150 | 0.192 | 0.235 | 0.219 |
|  | +2 | 0.097 | 0.183 | 0.142 | 0.175 | 0.136 | 0.137 |
|  | +3 | 0.148 | 0.198 | 0.148 | 0.173 | 0.204 | 0.218 |
|  | +4 | 0.002 | 0.019 | 0.004 | 0.010 | 0.022 | 0.013 |
|  | +5 | 0.037 | 0.061 | 0.041 | 0.047 | 0.049 | 0.121 |
| **ALA** | +0 | 0.939 | 0.890 | 0.819 | 0.755 | 0.719 | 0.714 |
|  | +1 | 0.050 | 0.086 | 0.137 | 0.186 | 0.211 | 0.195 |
|  | +2 | 0.011 | 0.024 | 0.044 | 0.060 | 0.070 | 0.091 |
| **ASP** | +0 | 0.941 | 0.906 | 0.835 | 0.707 | 0.603 | 0.552 |
|  | +1 | 0.049 | 0.073 | 0.123 | 0.199 | 0.262 | 0.294 |
|  | +2 | 0.008 | 0.017 | 0.033 | 0.070 | 0.098 | 0.105 |
|  | +3 | 0.002 | 0.004 | 0.009 | 0.025 | 0.037 | 0.049 |
| **CIT** | +0 | 0.843 | 0.794 | 0.712 | 0.603 | 0.509 | 0.423 |
|  | +1 | 0.080 | 0.104 | 0.137 | 0.169 | 0.201 | 0.202 |
|  | +2 | 0.065 | 0.082 | 0.108 | 0.136 | 0.152 | 0.139 |
|  | +3 | 0.006 | 0.011 | 0.022 | 0.043 | 0.066 | 0.073 |
|  | +4 | 0.004 | 0.007 | 0.015 | 0.030 | 0.040 | 0.041 |
|  | +5 | 0.000 | 0.001 | 0.002 | 0.009 | 0.017 | 0.022 |
|  | +6 | 0.001 | 0.001 | 0.003 | 0.008 | 0.014 | 0.100 |
| **Erytol** | +0 | 0.966 | 0.967 | 0.967 | 0.959 | 0.953 | 0.940 |
|  | +1 | 0.039 | 0.038 | 0.039 | 0.044 | 0.046 | 0.055 |
|  | +2 | 0.000 | 0.000 | 0.000 | 0.000 | 0.000 | 0.000 |
|  | +3 | 0.001 | 0.001 | 0.001 | 0.001 | 0.002 | 0.004 |
|  | +4 | 0.001 | 0.001 | 0.001 | 0.002 | 0.004 | 0.009 |
| **F6P C4-C6** | +0 | 0.802 | 0.812 | 0.782 | 0.772 | 0.770 | 0.744 |
|  | +1 | 0.081 | 0.074 | 0.092 | 0.097 | 0.095 | 0.104 |
|  | +2 | 0.064 | 0.056 | 0.057 | 0.057 | 0.061 | 0.056 |
|  | +3 | 0.053 | 0.058 | 0.069 | 0.074 | 0.074 | 0.096 |
| **F6P C1-C6** | +0 | 0.467 | 0.381 | 0.341 | 0.309 | 0.294 | 0.304 |
|  | +1 | 0.439 | 0.498 | 0.508 | 0.526 | 0.546 | 0.513 |
|  | +2 | 0.053 | 0.067 | 0.080 | 0.086 | 0.091 | 0.087 |
|  | +3 | 0.023 | 0.029 | 0.034 | 0.039 | 0.041 | 0.041 |
|  | +4 | 0.009 | 0.015 | 0.020 | 0.021 | 0.020 | 0.025 |
|  | +5 | 0.001 | 0.002 | 0.003 | 0.005 | 0.004 | 0.004 |
|  | +6 | 0.007 | 0.008 | 0.014 | 0.014 | 0.005 | 0.026 |
| **FBP** | +0 | 0.522 | 0.450 | 0.406 | 0.381 | 0.369 | 0.364 |
|  | +1 | 0.352 | 0.386 | 0.410 | 0.422 | 0.425 | 0.424 |
|  | +2 | 0.051 | 0.070 | 0.079 | 0.090 | 0.093 | 0.091 |
|  | +3 | 0.046 | 0.055 | 0.063 | 0.063 | 0.064 | 0.063 |
|  | +4 | 0.014 | 0.019 | 0.026 | 0.029 | 0.030 | 0.031 |
|  | +5 | 0.000 | 0.004 | 0.000 | -0.002 | 0.001 | 0.002 |
|  | +6 | 0.015 | 0.016 | 0.017 | 0.017 | 0.018 | 0.024 |
| **FUM** | +0 | 0.908 | 0.866 | 0.821 | 0.742 | 0.644 | 0.596 |
|  | +1 | 0.047 | 0.058 | 0.091 | 0.138 | 0.186 | 0.181 |
|  | +2 | 0.043 | 0.070 | 0.075 | 0.093 | 0.124 | 0.166 |
|  | +3 | 0.003 | 0.006 | 0.012 | 0.021 | 0.035 | 0.041 |
|  | +4 | -0.001 | 0.000 | 0.002 | 0.006 | 0.012 | 0.015 |
| **G6P C3-C6** | +0 | 0.832 | 0.800 | 0.776 | 0.760 | 0.756 | 0.745 |
|  | +1 | 0.085 | 0.104 | 0.118 | 0.124 | 0.126 | 0.126 |
|  | +2 | 0.026 | 0.028 | 0.032 | 0.034 | 0.035 | 0.036 |
|  | +3 | 0.015 | 0.022 | 0.025 | 0.030 | 0.031 | 0.030 |
|  | +4 | 0.042 | 0.046 | 0.050 | 0.051 | 0.053 | 0.064 |
| **G6P C1-C6** | +0 | 0.450 | 0.359 | 0.301 | 0.287 | 0.280 | 0.275 |
|  | +1 | 0.449 | 0.503 | 0.543 | 0.546 | 0.545 | 0.545 |
|  | +2 | 0.046 | 0.064 | 0.074 | 0.079 | 0.082 | 0.083 |
|  | +3 | 0.019 | 0.026 | 0.029 | 0.033 | 0.035 | 0.034 |
|  | +4 | 0.008 | 0.014 | 0.017 | 0.020 | 0.020 | 0.020 |
|  | +5 | 0.002 | 0.003 | 0.004 | 0.005 | 0.005 | 0.005 |
|  | +6 | 0.025 | 0.031 | 0.032 | 0.029 | 0.033 | 0.038 |
| **GLC** | +0 | 0.949 | 0.921 | 0.901 | 0.870 | 0.855 | 0.828 |
|  | +1 | 0.047 | 0.055 | 0.064 | 0.081 | 0.082 | 0.105 |
|  | +2 | -0.003 | 0.004 | 0.002 | 0.007 | 0.006 | 0.010 |
|  | +3 | -0.003 | -0.002 | 0.005 | 0.010 | 0.012 | 0.014 |
|  | +4 | 0.010 | 0.021 | 0.028 | 0.032 | 0.044 | 0.043 |
| **GLU** | +0 | 0.936 | 0.910 | 0.844 | 0.709 | 0.562 | 0.453 |
|  | +1 | 0.049 | 0.060 | 0.090 | 0.149 | 0.223 | 0.276 |
|  | +2 | 0.013 | 0.027 | 0.056 | 0.109 | 0.148 | 0.162 |
|  | +3 | 0.001 | 0.002 | 0.008 | 0.026 | 0.051 | 0.059 |
|  | +4 | 0.001 | 0.001 | 0.002 | 0.007 | 0.016 | 0.051 |
| **LEU** | +0 | 0.863 | 0.741 | 0.586 | 0.470 | 0.427 | 0.552 |
|  | +1 | 0.124 | 0.221 | 0.315 | 0.339 | 0.324 | 0.239 |
|  | +2 | 0.010 | 0.029 | 0.072 | 0.127 | 0.160 | 0.119 |
|  | +3 | 0.001 | 0.007 | 0.023 | 0.051 | 0.069 | 0.053 |
|  | +4 | 0.000 | 0.001 | 0.004 | 0.009 | 0.016 | 0.015 |
|  | +5 | 0.002 | 0.001 | 0.001 | 0.003 | 0.004 | 0.023 |
| **MAL** | +0 | 0.918 | 0.879 | 0.800 | 0.669 | 0.550 | 0.484 |
|  | +1 | 0.057 | 0.079 | 0.124 | 0.190 | 0.245 | 0.279 |
|  | +2 | 0.020 | 0.030 | 0.052 | 0.092 | 0.127 | 0.140 |
|  | +3 | 0.004 | 0.009 | 0.019 | 0.035 | 0.055 | 0.061 |
|  | +4 | 0.001 | 0.003 | 0.005 | 0.014 | 0.023 | 0.036 |
| **Manol** | +0 | 0.954 | 0.954 | 0.947 | 0.932 | 0.906 | 0.865 |
|  | +1 | 0.046 | 0.046 | 0.051 | 0.063 | 0.083 | 0.111 |
|  | +2 | 0.000 | -0.001 | 0.000 | 0.002 | 0.004 | 0.007 |
|  | +3 | 0.000 | 0.000 | 0.000 | 0.001 | 0.003 | 0.005 |
|  | +4 | 0.000 | 0.000 | 0.001 | 0.002 | 0.005 | 0.011 |
| **PEP** | +0 | 0.805 | 0.783 | 0.716 | 0.671 | 0.694 | 0.674 |
|  | +1 | 0.132 | 0.144 | 0.184 | 0.201 | 0.205 | 0.207 |
|  | +2 | 0.026 | 0.028 | 0.038 | 0.047 | 0.029 | 0.034 |
|  | +3 | 0.038 | 0.045 | 0.063 | 0.081 | 0.072 | 0.085 |
| **2PG** | +0 | 0.794 | 0.739 | 0.696 | 0.687 | 0.673 | 0.689 |
|  | +1 | 0.136 | 0.171 | 0.196 | 0.202 | 0.214 | 0.214 |
|  | +2 | 0.026 | 0.035 | 0.048 | 0.037 | 0.039 | 0.027 |
|  | +3 | 0.044 | 0.055 | 0.060 | 0.074 | 0.075 | 0.070 |
| **3PG** | +0 | 0.804 | 0.758 | 0.714 | 0.697 | 0.688 | 0.680 |
|  | +1 | 0.137 | 0.170 | 0.199 | 0.206 | 0.208 | 0.214 |
|  | +2 | 0.022 | 0.025 | 0.034 | 0.037 | 0.039 | 0.034 |
|  | +3 | 0.037 | 0.047 | 0.054 | 0.060 | 0.066 | 0.072 |
| **PYR** | +0 | 0.932 | 0.943 | 0.897 | 0.858 | 0.834 | 0.892 |
|  | +1 | 0.056 | 0.045 | 0.079 | 0.111 | 0.121 | 0.075 |
|  | +2 | 0.003 | 0.002 | 0.005 | 0.003 | 0.015 | 0.014 |
|  | +3 | 0.009 | 0.010 | 0.019 | 0.028 | 0.029 | 0.019 |
| **R5P** | +0 | 0.877 | 0.848 | 0.832 | 0.819 | 0.818 | 0.822 |
|  | +1 | 0.060 | 0.078 | 0.084 | 0.094 | 0.095 | 0.096 |
|  | +2 | 0.022 | 0.025 | 0.027 | 0.027 | 0.027 | 0.025 |
|  | +3 | 0.041 | 0.049 | 0.057 | 0.059 | 0.060 | 0.058 |
| **Arabtol** | +0 | 0.959 | 0.969 | 0.966 | 0.967 | 0.961 | 0.949 |
|  | +1 | 0.035 | 0.034 | 0.038 | 0.034 | 0.038 | 0.046 |
|  | +2 | 0.000 | -0.004 | -0.007 | -0.005 | -0.004 | -0.001 |
|  | +3 | 0.000 | 0.000 | -0.001 | 0.002 | 0.001 | 0.001 |
|  | +4 | 0.006 | 0.002 | 0.003 | 0.003 | 0.004 | 0.006 |
| **SED7P** | +0 | 0.865 | 0.813 | 0.778 | 0.759 | 0.752 | 0.744 |
|  | +1 | 0.077 | 0.107 | 0.126 | 0.136 | 0.138 | 0.141 |
|  | +2 | 0.017 | 0.025 | 0.029 | 0.031 | 0.032 | 0.028 |
|  | +3 | 0.013 | 0.021 | 0.028 | 0.032 | 0.034 | 0.033 |
|  | +4 | 0.027 | 0.035 | 0.039 | 0.042 | 0.044 | 0.053 |
| **SER** | +0 | 0.950 | 0.911 | 0.846 | 0.777 | 0.726 | 0.798 |
|  | +1 | 0.043 | 0.072 | 0.127 | 0.186 | 0.233 | 0.161 |
|  | +2 | 0.007 | 0.016 | 0.026 | 0.037 | 0.041 | 0.040 |
| **SUC** | +0 | 0.861 | 0.899 | 0.829 | 0.756 | 0.698 | 0.745 |
|  | +1 | 0.047 | 0.062 | 0.085 | 0.133 | 0.194 | 0.176 |
|  | +2 | 0.014 | 0.022 | 0.034 | 0.069 | 0.107 | 0.079 |
|  | +3 | 0.004 | 0.017 | 0.052 | 0.042 | 0.000 | 0.000 |
|  | +4 | 0.074 | 0.000 | 0.000 | 0.000 | 0.000 | 0.000 |
| **TRE** | +0 | 0.940 | 0.936 | 0.926 | 0.900 | 0.848 | 0.709 |
|  | +1 | 0.059 | 0.062 | 0.070 | 0.090 | 0.129 | 0.168 |
|  | +2 | 0.000 | 0.001 | 0.001 | 0.005 | 0.011 | 0.017 |
|  | +3 | 0.000 | 0.000 | 0.001 | 0.002 | 0.004 | 0.007 |
|  | +4 | 0.001 | 0.001 | 0.001 | 0.001 | 0.003 | 0.004 |
|  | +5 | 0.000 | 0.000 | 0.000 | 0.000 | 0.001 | 0.004 |
|  | +6 | 0.000 | 0.000 | 0.001 | 0.003 | 0.006 | 0.092 |
| **VAL** | +0 | 0.932 | 0.903 | 0.856 | 0.800 | 0.785 | 0.760 |
|  | +1 | 0.056 | 0.076 | 0.104 | 0.136 | 0.143 | 0.118 |
|  | +2 | 0.008 | 0.017 | 0.032 | 0.051 | 0.056 | 0.046 |
|  | +3 | 0.002 | 0.003 | 0.006 | 0.010 | 0.013 | 0.013 |
|  | +4 | 0.001 | 0.002 | 0.002 | 0.003 | 0.004 | 0.063 |
